# Micromachined Resonators: A Review

**DOI:** 10.3390/mi7090160

**Published:** 2016-09-08

**Authors:** Reza Abdolvand, Behraad Bahreyni, Joshua E. -Y. Lee, Frederic Nabki

**Affiliations:** 1 Dynamic Microsystems Lab, Department of Electrical and Computer Engineering, University of Central Florida, Orlando, FL 32816, USA; reza@ece.ucf.edu; 2 School of Mechatronic Systems Engineering, Simon Fraser University, Surrey, BC V3T 0A3, Canada; 3 State Key Laboratory of Millimeter Waves, City University of Hong Kong, Kowloon, Hong Kong, China; josh.lee@cityu.edu.hk; 4 Department of Electrical Engineering, École de Technologie Supeérieure, Montreal, QC H3C 1K3, Canada; frederic.nabki@etsmtl.ca

**Keywords:** resonance, microresonators, micro-electro-mechanical systems

## Abstract

This paper is a review of the remarkable progress that has been made during the past few decades in design, modeling, and fabrication of micromachined resonators. Although micro-resonators have come a long way since their early days of development, they are yet to fulfill the rightful vision of their pervasive use across a wide variety of applications. This is partially due to the complexities associated with the physics that limit their performance, the intricacies involved in the processes that are used in their manufacturing, and the trade-offs in using different transduction mechanisms for their implementation. This work is intended to offer a brief introduction to all such details with references to the most influential contributions in the field for those interested in a deeper understanding of the material.

## 1. Introduction

Microelectromechanical systems (MEMS) are a disruptive technology, much like lasers or integrated circuits. As such, MEMS have an overarching applicability and impact in several sectors such as telecommunications, consumer electronics, transportation, building automation and healthcare. The MEMS market is expected to sustain continued growth made possible by many technological revolutions fueled by, among others, the Internet of Things and wearable electronics, and it is expanding at an increasing rate, projected to almost double from $11B in 2014 to $21B in 2020 with MEMS resonator representing a growing market share [1].

The concept of MEMS resonators, mechanically resonating micro-structures that are electrically brought into resonance, along with some of their advantages and applications were introduced in their early form in the 1960s [2]. Today, amidst the widespread use of MEMS, MEMS resonators are generating significant research and commercial interest, and are poised to capture a significant portion of the MEMS market because of their numerous large volume and high impact applications. These include sensing applications, where changes in a resonant element are used to monitor a given quantity [3], timing applications, where a resonant element is used within an electronic system to generate a high quality clock signal [4], or in filtering applications, where resonant structures implement filters that can be of use in radiofrequency wireless transceivers [5]. MEMS resonators are expected to become prevalent in these applications because they are well-suited to low-cost batch fabrication, being manufactured with fabrication techniques similar to those widespread in integrated circuit manufacturing. Moreover, unlike other resonant elements such as quartz crystals, MEMS resonators have the potential for higher levels of integration with microelectronics at the die or package level [6]. These advantages can lead to reduced cost and form-factor systems that can have enhanced performance and more functionality. However, before MEMS resonators can completely replace other types of resonant elements, some challenges remain such as material limitations, temperature stability, packaging or batch integration with electronics.

MEMS resonators have been the subject of several reviews that covered various aspects of the field from the devices themselves to their various applications, e.g., [7,8,9,10,11,12]. This paper is aimed at surveying a wide range of topics and prior work related to MEMS resonators in order to provide readers with a better understanding of their operation and to give an overview of their evolution over the last thirty years towards achieving their foreseen potential, among which their penetration of the aforementioned applications. Specifically, the paper first details the operating principles of MEMS resonators, covering modeling, properties, resonance modes, damping mechanisms and transduction mechanisms. It then discusses techniques and challenges behind the manufacturing of MEMS resonators, touching on materials and processes that have been used in their fabrication, including their fabrication using complementary metal oxide semiconductor (CMOS) processes. Current and emerging applications of MEMS resonators, namely their use in timing, sensing and radio-frequency systems are then described. This includes an overview of the operating principles, performance metrics, design considerations and latest developments in MEMS resonator-based oscillators, a key MEMS resonator-based block suitable for sensing and timing applications. Where appropriate, the paper surveys the latest research developments and directions pertaining to MEMS resonators and discusses them. In addition, it provides information on the design and the use of MEMS resonators standalone and within systems.

## 2. Basic Model and Properties

**Figure 1:**
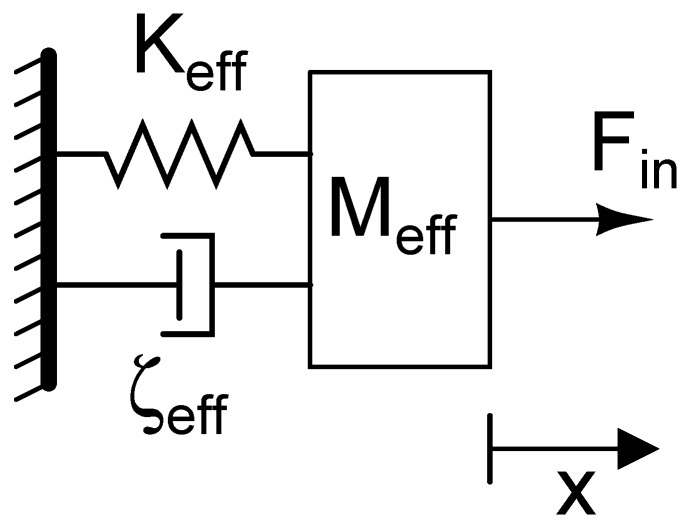
A mass-spring-damper system.

In a vibrating mechanical system, the kinetic and potential energies are continuously converted to each other. Most systems exhibit a frequency dependent response where this transfer of energy is optimum at certain frequencies (i.e., losses are minimum), known as the *resonant frequencies* of the system. For low enough damping, the system response shows peaks at these particular frequencies. Additionally, each resonant frequency corresponds to a particular pattern of motion for the components of the mechanical system which is known as a *mode shape*. To exhibit resonance, a mechanical system must possess the capacity to store both kinetic and potential energies. Therefore, the basic resonator structure is a mass-spring system. In physical systems, additionally, there are always energy loss mechanisms. A simple mode for mechanical losses is a damper. This combination of mass-damper-spring system represents the simplest model for a resonator, as shown in Figure 1. Using Newton’s laws of motion, the relationship between the displacements of the mass and input force can be found from:

(1) $$M_{eff}\frac{\partial^{2}x}{\partial t^{2}}+\zeta_{eff}\frac{\partial x}{\partial t}+K_{eff}x=F_{in}$$

where $F_{in}$ is the input force, $M_{eff}$ is the effective mass of the system, $K_{eff}$ is the effective stiffness, and $\zeta_{eff}$ represents the effective total losses in the system. The system transfer function is given by

(2) $$H\left(s\right)=\frac{X\left(s\right)}{F_{in}\left(s\right)}=\frac{1}{M_{eff}s^{2}+\zeta_{eff}s+K_{eff}}=\frac{1}{K_{eff}}\left(\frac{\omega_{0}^{2}}{s^{2}+\frac{\omega_{0}}{Q}s+\omega_{0}^{2}}\right)$$

where s is the complex frequency, $\omega_{0}$ is the undamped resonant frequency of the system (i.e., natural frequency) and Q is the quality factor. For a second order system, the undamped resonant frequency is:

(3) $$\omega_{0}=2\pi f_{0}=\sqrt{\frac{K_{eff}}{M_{eff}}}$$

The quality factor is defined as:

(4) $$Q=2\pi \frac{\text{Average energy stored}}{\text{Energy lost per cycle}}$$

Parameters $\omega_{0}$ (or $f_{0}$) and Q are the two significant performance metrics in the microresonator domain. Due to their sizes, the resonant frequencies of microdevices are typically in kHz to MHz range but can be in the GHz range for properly designed devices [13,14,15]. For a device that is intended to be used as a microresonator, Q ranges from thousands to millions depending on the operating conditions and device design [16,17,18]. Many applications benefit from maximizing both the resonant frequency and quality factor of a resonator even though there is some trade-off between the two. Consequently, their product, $f_{0}\cdot Q$, is a common figure of merit stated for resonators [19,20].

The relationship between the resonant frequency, $\omega_{r}$, and the undamped natural frequency, $\omega_{0}$, of a second order system is [21]:

(5) $$\omega_{r}=\omega_{0}\sqrt{1-\frac{1}{2\;Q^{2}}}$$

**Figure 2:**
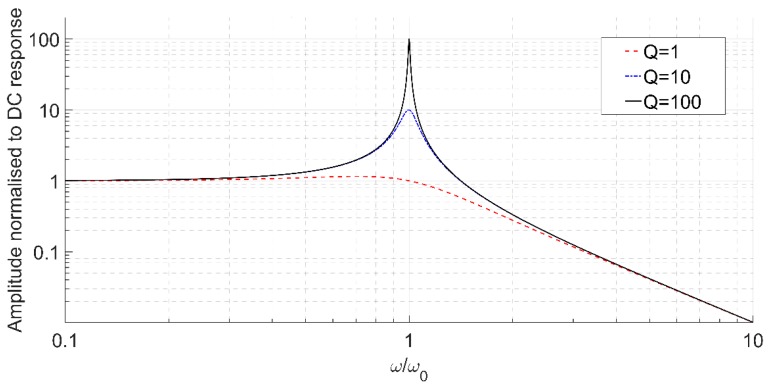
Time and frequency response of resonant systems.

It can be seen that, for large quality factors, as is the case for most micromachined resonators, $\omega_{r}\approx \omega_{0}$. By running a single frequency response measurement, one can estimate the resonant frequency of a microresonator by locating the peak in the frequency response while the quality factor (for Q≫1) can be estimated from:

(6) $$Q=\frac{f_{0}}{\Delta f_{-3dB}}=\frac{\omega_{0}}{2}\frac{d∢H\left(\omega \right)}{d\omega }$$

where $\Delta f_{-3dB}$ is the −3dB bandwidth around the resonant frequency. In addition to a narrower bandwidth, high-Q systems exhibit a higher peak amplitude at resonance that is Q times the low frequency response (see Figure 2).

While the basic second-order model of the resonator is quite useful to study a device response near its resonant frequency, it is often rather simplistic. Most mechanical systems, even those composed of discrete components, have numerous different mode shapes and corresponding resonant frequencies. If the resonant frequencies are far from each other, the device response can be analyzed using the basic mass-spring-damper method around each resonant frequency. Otherwise, a higher order model needs to be constructed that can potentially include the coupling between different modes. Some continuous mechanical systems can be broken into simpler subsystems, allowing for the treatment of the system as a lumped one. This is particularly helpful when it is possible to estimate concentrated masses and the effective stiffnesses of bodies that connect them to each other within the structure. In many cases, however, the whole system needs to be treated as a distributed mass-spring system. Dynamics of such systems is studied using the acoustic wave propagation models and theories [22].

Distributed systems, in theory, have infinite mode frequencies and shapes. In practice, however, a limited number of these modes need to be studied in a frequency band of interest. The basic spring-mass-damper model can once again be used if one estimates the effective mass and stiffness of the system for the particular mode shape of interest. Rayleigh’s method is a fairly robust and yet simple technique to estimate the effective mass and stiffness of a system once good estimates for the mode shape of the device are available [23,24]. If the losses can be ignored, the resonant frequency of the *n*th mode of the system can be found from:

(7) $$\omega_{n}^{2}=\frac{{\vec{\left[x\right]}}^{\prime }\left[K\right]\vec{\left[x\right]}}{{\vec{\left[\dot{x}\right]}}^{\prime }\left[ℳ\right]\vec{\left[\dot{x}\right]}}$$

where [K] and [ℳ] are the stiffness and mass matrices for the system and $\vec{\left[x\right]}$ and $\vec{\left[\dot{x}\right]}$ are the displacement and velocity vectors for the *n*th mode shape of interest, respectively.

## 3. Electric Circuit Representation

In a typical microresonator application, the micromechanical structure is forced into vibrations by converting an input electrical signal into a force and applying it to the device. Vibrations of the structure are then picked up and often converted back to the electrical domain through various transduction techniques. Consequently, from the point of view of interrogating instruments, the device is assumed electrical. On the other hand, it is common to use the analogy between electrical and mechanical resonators to build an equivalent electrical circuit for a micromachined resonator [21,24,25,26]. Such a model is often built from a set of experimental measurements and then is used in electric circuit simulators. This approach is particularly useful if the resonator needs to be modelled with the drive or sense electronics, allowing for co-simulation of the entire system within the electrical domain. To represent a mechanical device with electrical elements, proper mapping of mechanical to electrical quantities is needed. A common set of mapping rules is summarized in Table 1 [24,25,26].

**Figure 3:**
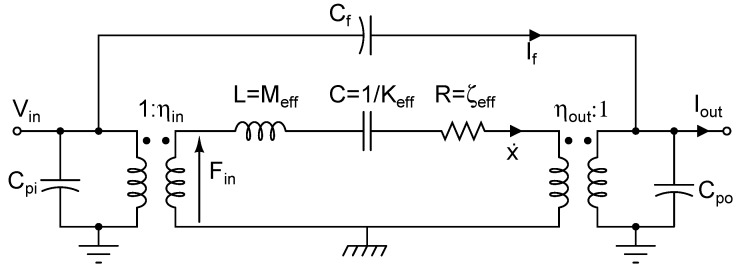
Equivalent electrical representation of an electrostatic resonator including the feedthrough capacitor $C_{f}$ and parasitic capacitors $C_{pi}$ and $C_{po}$.

In most cases, a resonant device is modelled as a series Resistance-Inductance-Capacity (RLC) circuit. The transductions from the electrical to mechanical domain and vice versa are modelled with transformers with proper winding ratios or controlled voltage or current sources. Other elements, especially parasitic and feedthrough capacitors, may be added to the equivalent circuit so that the model produces results similar to experimental measurements. Figure 3 illustrates an equivalent electrical model for a microresonator with electrostatic input and output ports. The transformer at the input port converts an input voltage to a force and applies it to the mechanical system represented by the series RLC circuit. At the output, another transformer converts velocities of the mechanical structure back to an electrical current. Similar models can be developed for other transduction mechanisms such as piezoelectric or thermal devices considering the mechanisms involved in converting the electrical signal to a mechanical one and vice versa. In all cases, the electromechanical coupling coefficients, $\eta_{\text{in}}$ and $\eta_{\text{out}}$, need to be defined according to the employed transduction mechanism. It is common practice to simplify the model further by removing the transformers and scaling the equivalent circuit values accordingly. Note the inclusion of the feedthrough capacitance in the model. This parasitic capacitance in many cases poses a challenge in a proper measurement of resonator response as the feedthrough current that travels through it can be significantly larger than the current produced by the resonator.

### Modelling of Nonlinearities

There can be several sources of nonlinearities in micromechanical resonators [27,28,29,30,31,32,33]. Elastic properties of most materials are a function of stresses applied to them. Even brittle materials such as silicon exhibit some stress-dependent behavior under large stresses [34]. On the other hand, internal stresses produced from large displacements can alter the stiffness of the structure, as is the case for the stiffening of beams under large loads. Both of these phenomena affect the dynamic response of the device. In many cases, for instance where electrostatic or thermal actuators are used, the actuation mechanism itself is inherently nonlinear. Even when the actuation mechanism is linearized for small displacements, for example by adding a large DC signal to the AC actuation signal in electrostatic resonators, there can be other sources of nonlinearities. For example, the electrostatic force produced by a voltage applied between two parallel electrodes can be found from:

(8) $$F_{e}=\frac{1}{2}\nabla CV^{2}$$

where ∇C is the gradient of the capacitance between the two electrodes and V is the voltage applied between them. While the nonlinear dependence on input voltage is apparent, in many cases the gradient term is a function of the separation between the electrodes, and hence can vary nonlinearly as the two electrodes move with respect to each other [29].

A well-known consequence of nonlinearities is a jump phenomenon in the frequency response of the device as the resonant frequency of the device will depend on the signal amplitude. This phenomenon is known as bifurcation. If the device response is studied through frequency sweeps, one will observe different device responses for upward or downward sweeps. For nonlinearities that increase the stiffness of the structure, the resonant frequency shifts up while those which soften the structure (e.g., electrostatic nonlinearities), the resonant frequency moves towards lower frequencies [35]. Nonlinearities in resonators has been studied extensively for macro- and micro-scale device [21,29,36,37]. The resonator nonlinearities are modelled by adding higher order terms to the effective spring constant of the structure and solving the resulting nonlinear equation of motions. Perturbation analysis is often employed to analyze the behavior of the systems with small nonlinearities. It has been shown that the total nonlinear behavior of the system can be modified by taking advantage of the different, and sometimes opposite, interactions between different mechanisms of nonlinearity [38,39].

## 4. Resonance Modes

### 4.1. Flexural Modes

**Figure 4:**
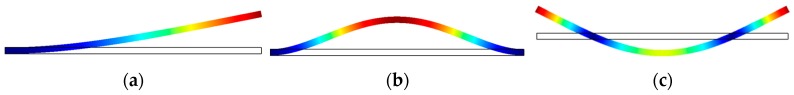
Common flexural mode beams classified according boundary conditions: (**a**) cantilever, β = 0.16154; (**b**) clamped-clamped beam, β = 1.02792; (**c**) free-free beam, β = 1.02792.

Flexural mode vibrations are characterized by bending of the structure along its length (*l*) such that the motion in the transverse direction perpendicular to the length. Flexural modes can be excited in both beam and plate structures. In the case of beams, the motion could be within the plane of fabrication (i.e., along its width w) or out of plane (i.e., along its thickness t). For beam structures, the vibration mode shape is determined by the boundary conditions applied to the structure. Various examples of flexural mode shapes are illustrated in Figure 4 along with the corresponding boundary conditions applied to the beam. The resonant frequency of a given mode for a beam resonator of length *L* and thickness *t* vibrating out of plane can be generalized according to the following formula:

(9) $$f=\beta \left(\frac{t}{L^{2}}\right)\sqrt{\frac{E}{\rho }}$$

where β is a dimensionless coefficient that is determined by the shape of the vibration mode, which in turn is depends on the respective boundary conditions applied to the structure. Equation (9) assumes that the beam is fabricated with one material whereby *E* denotes the Young’s modulus and ρ is the density. As can be seen from Equation (9), the resonant frequency is independent of the beam width when it is vibrating in the thickness direction. Figure 4 summarizes the most common flexural modes based on beam structures, classified according to the boundary conditions applied. The corresponding values of β have been referenced from [40].

As depicted in Figure 4a, a cantilever such as the ones reported in [41,42,43,44,45] is defined by a beam that is clamped at one end and free on the other such that the maximum deflection takes place at the tip of the beam furthest away from the clamped end. As illustrated in Figure 4b the clamped-clamped or doubly-clamped beam such as the ones reported in [46,47,48,49] is defined by both ends of the structure clamped such that the maximum deflection takes place at mid-length. Both cantilever and clamped-clamped beams are commonly adopted structures for mass sensing applications due to their structural simplicity and potential for realizing small proof masses [50,51]. Finally, as shown in Figure 4c it is also possible to realize a flexural mode free-free beam whereby both ends are free [52]. The beam here is clamped at two positions along the length where the deflection is zero [18].

**Figure 5:**
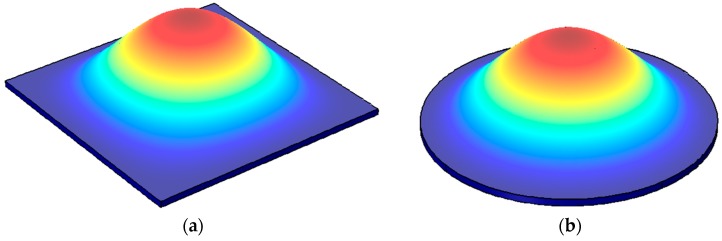
Common flexural mode membrane resonators: (**a**) square membrane; (**b**) circular membrane.

Figure 5 illustrates the fundamental modes observed in membrane structures, most of which are square or circular. The edges of the membranes are clamped and the maximum deflection occurs at the center of the membrane. The resonant frequencies of plate structures follow the form of the Equation (9). Membrane resonators are commonly used to implement micromachined ultrasonic transducers (MUTs) [53,54].

### 4.2. Bulk Modes

**Figure 6:**
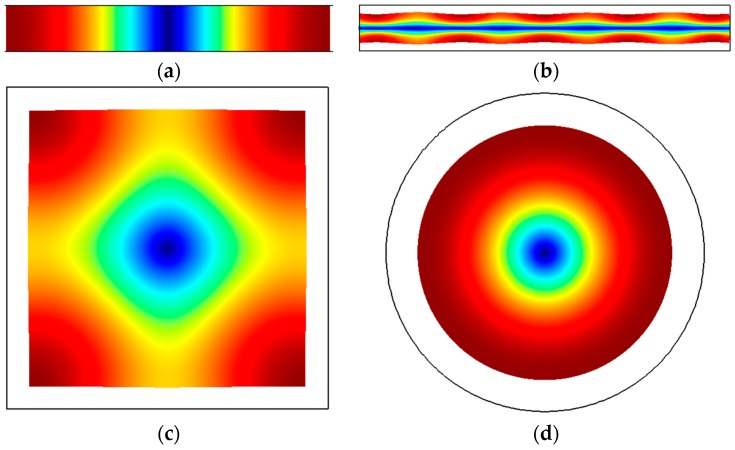
Bulk mode shapes based on rectangular plates, square plates, and circular disks: (**a**) length-extensional (LE) mode, β = 1, λ = 2L; (**b**) width-extensional (WE) mode, β = 1, λ = 2W; (**c**) square-extensional (SE) mode, β = 1, λ = 2L; (**d**) radial breathing mode, β = 1, λ = 2R.

**Figure 7:**
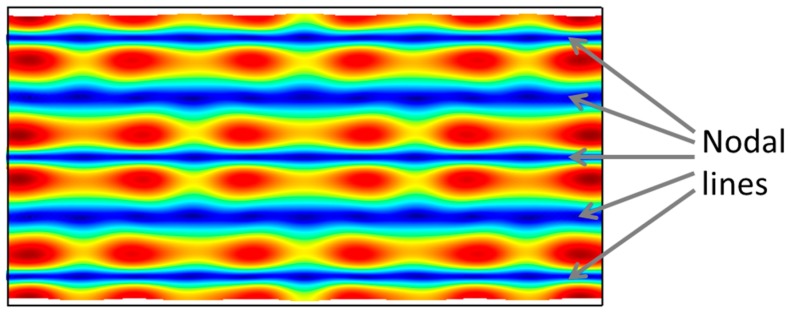
Displacement profile of the 5th order width-extensional (WE) mode of vibration.

In contrast to flexural mode resonators, bulk mode resonators are characterized by deformation of the structure through planar expansions or contractions rather than bending. In terms of geometrical dependence, the resonant frequencies of bulk modes only depend on the lateral physical dimensions of the structure (e.g., width or length). In other words, the lateral features of the structure alone determine the acoustic wavelength (λ) of the vibration mode. As such, the resonant frequencies of bulk mode resonators can be generalized by the following form:

(10) $$f_{bulk}=\frac{\beta }{\lambda }\sqrt{\frac{E_{bulk}}{\rho }}$$

where *E_bulk_* is the effective modulus of the plate structure defined for a given axis of motion. Bulk modes resonators have been reported for beams [55,56,57], rectangular plates [58,59], square plates [60,61], and circular disks [62,63]. In the case of bars/beams, *E_bulk_* simplifies to the Young’s modulus. In comparison to flexural mode resonators, bulk mode resonators are much stiffer for the same physical dimension scales. This in turn translates to higher frequencies for the same physical dimensions. As such, bulk mode resonators are favored over flexural mode resonators for higher frequency applications owing to their more efficient frequency-to-size scaling characteristic. The mode shapes of various examples of bulk mode resonators reported in the literature are illustrated in Figure 6. In the case of bulk modes, the standing waves in the solid structures are longitudinal waves. It can also be seen that every part of the structure undergoes either compression or expansion apart from the center. The center of the structure is the most obvious choice to clamp the structure wherever possible from the viewpoint of the fabrication from the perspective of minimizing losses to the supports. It should be noted that the lateral bulk modes described in Figure 3 can each be excited at higher order modes of vibration. This is rather commonly the case for the length-extensional (LE) and width-extensional (WE) modes. As an example, the 5th order mode of the WE mode of vibration is illustrated in Figure 7, which can be described as having 5 nodal lines. Higher order modes are particularly common in the case of piezoelectric resonators [64]. Lateral bulk modes of resonance, particularly when applied to piezoelectric resonators, are referred to as contour modes [65] wherein the acoustic radiation patterns are viewed as contours in the plane of fabrication. This is in contrast to thickness vibration modes that are typically viewed by devices such as the film bulk acoustic resonator (FBAR) [66].

### 4.3. Shear Modes

Shear mode resonators are similar to bulk mode resonators in that their acoustic wavelength is also determined only by the lateral features of the structure. However, in contrast to bulk modes, shear modes are defined by shear waves instead of longitudinal waves. As such, their stiffness constants are defined by the shear modulus of the structural material rather than the Young’s modulus. Therefore, the resonator frequency of lateral shear modes is given by the shear modulus *G*, instead of the Young’s modulus:

(11) $$f_{shear}=\frac{\beta }{\lambda }\sqrt{\frac{G}{\rho }}$$

**Figure 8:**
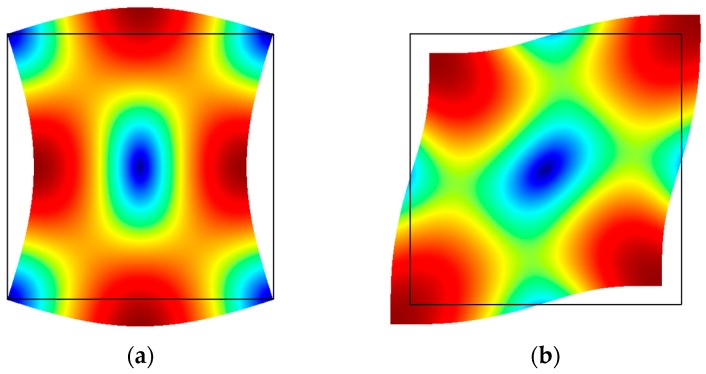
Lateral shear modes based on a square plate resonator: (**a**) Lamé mode , β = 1, λ = √2L; (**b**) FS mode, β = 1.283, λ = 2L.

This feature of being defined by shear is evident when one considers the mode shapes. In square plates, shear modes that have been observed include the Lamé [67,68,69] and face shear (FS) [70] modes, which are depicted in Figure 8a,b respectively. In both cases, the direction of motion is always equal and opposite between two orthogonal axes within the plane of fabrication. In other words, while the structure is defined by expansion in one axis, it is simultaneously defined by contraction in the orthogonal axis. In every part of the plate, the in-plane strain components are equal and opposite, thereby cancelling each other out when summed up. As such, the volumetric change is theoretically zero everywhere across the square plate. This isochoric property leads to the interesting feature of lateral shear modes having theoretically no thermoelastic damping (TED). TED arises from irreversible heat flow between regions of expansion and contraction, and since there is no volume change during the operation of the resonator TED is thus zero in principle. Low TED allows for resonators with high quality factors in the millions. Consequently, adding holes in the structure for the purpose of fabrication breaks the isochoric property and introduces TED, resulting in a substantial drop in quality factor [71,72]. The shear wave appears 45° to the primary axis of deformation. In either case of the Lamé and FS modes, nodes appear at specific points along the edges of the square plate, where the structure can be conveniently clamped to minimize support losses where the displacement is zero but there is some rotation. For elastically anisotropic materials like single-crystal silicon, the relevant shear modulus is given by the axis of the shear wave.

**Figure 9:**
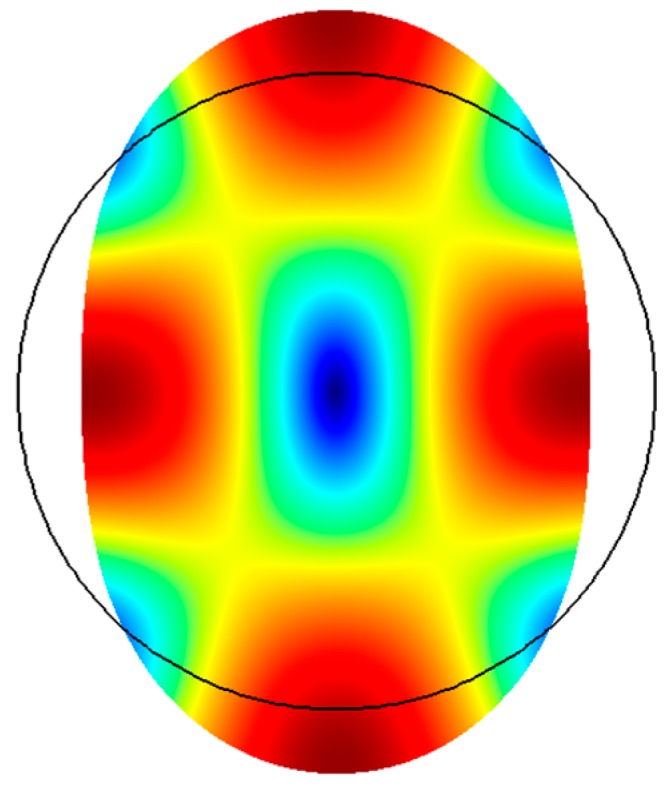
Wine glass mode observed in a circular disk resonator.

Shear modes have also been realized in circular disks as shown in Figure 9, from which we can see once again that the motion in one axis is equal and opposite to the orthogonal axis. This is commonly referred to as the wine glass mode [73,74]. Given the symmetry of the structure, the same elliptical mode shape can occur in two axes 45° apart. In isotropic solids, the two modes share the same frequency, which is known as mode degeneracy. In anisotropic solids, the two modes occur at slightly different frequencies [75].

### 4.4. Torsional Modes

**Figure 10:**
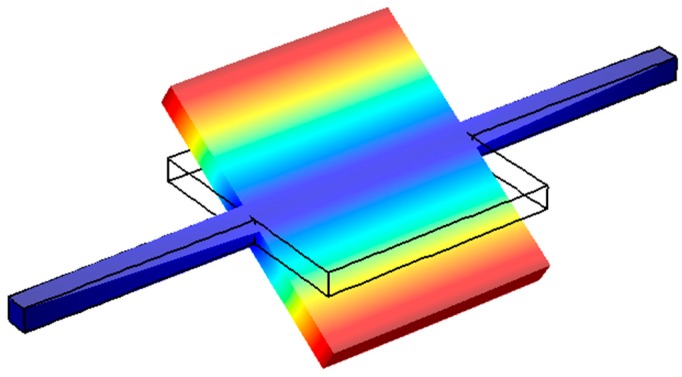
A torsional mode paddle resonator.

Torsional mode resonators are most typically found in the form of paddle resonators, which comprise a plate that is supported on two opposite ends by beams. The paddle resonator oscillates by means of rotating about the axis along which the supporting beams lie as illustrated in Figure 10. The supporting beams are clamped at the ends and experience a twisting motion as the plate oscillates about the axis of rotation. The beams undergoing torsion thus form the spring of the resonator and thereby define the spring constant while the rotating plate approximates to a rigid body that defines the proof mass of the resonator [76]. Torsional mode paddle resonators have been applied to sensing applications that include electrometers [77] and magnetometers [78].

### 4.5. Coupled Resonators

**Figure 11:**
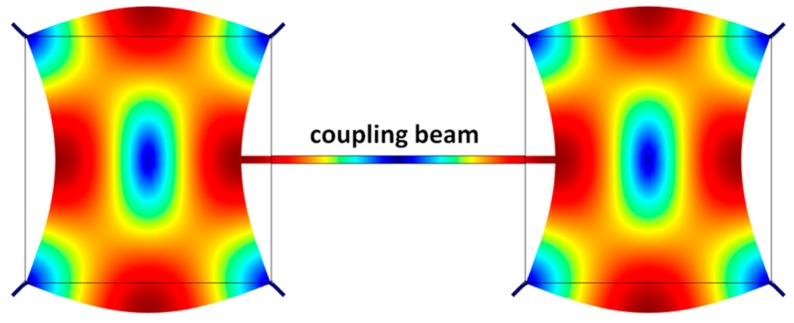
Array of Lamé mode resonators realized through mechanical coupling.

The above examples of this section have included only single resonators so far. Single resonators can in turn be mechanically coupled to realize an array of identical resonators. Assuming the same vibration mode for each resonator, the number of modes possible in an array increases with the size of the array. If the resonators are coupled to each other strongly, the frequency separation between the modes gets widened. This approach is particularly favorable for the purpose of increasing the output signal strength of MEMS resonators by creating arrays of the same resonator, synchronized to vibrate at the same frequency [79]. This is particularly useful in lowering the insertion loss of filters [80,81] as well as reducing the phase noise of MEMS oscillators [82,83]. Strong mechanical coupling has been demonstrated using coupling structures with lengths that are multiples of the acoustic half-wavelength (i.e., $\frac{n\lambda }{2}$, where *n* = 1, 2, 3,…) [84,85]. An illustration of an array of square plate resonators mechanically coupled together for synchronized oscillation is provided in Figure 11. Note that all the resonators in the array are vibrating in the Lamé mode and the phase between the resonators are the same [85]. While strong coupling pushes the modes apart, weak coupling results in closely separate modes, such as in defining a narrow passband in filters [86]. Weak mechanical coupling is similarly achieved by using coupling structures with lengths that are odd multiples of a quarter of the acoustic wavelength (i.e., $\frac{2n-1}{4}\lambda$, where *n* = 1, 2, 3,…) [80]. Alternatively, the electrostatic spring tuning effect that arises from the nonlinearity in a capacitive gap transducer can be used to realize a weak spring that is a function of voltage across the transducer. This electrostatic spring is used as the mechanical coupling element between the resonators [87]. Tuning the voltage across the transducer changes the coupling spring, which in turn tunes the separation of the passband [88]. Weakly coupled resonators are particularly interesting for sensing applications through exploiting mode localization, which involves manipulation of energy between two coupled modes [89]. This approach has been found to be beneficial for enhancing sensitivity by a few orders of magnitude [90] in comparison to conventional resonant sensing that depends on the perturbation of frequency while at the same time rejecting common-mode effects [91].

### 4.6. Other Modes

Based upon the WE mode, modifications to the geometry of the resonator have been made with the aim of concentrating the acoustic energy towards the center of the bulk mode resonator in order to reduce the energy distributed at the clamped ends of the resonator. Reducing the energy at the clamped ends of the resonator ultimately reduces leakage of energy to the substrate, thereby improving the quality factor of the resonator. Modifications with the aim of acoustic engineering include curving of the free edges of the resonator [92] or introducing steps [93].

## 5. Damping

A portion of the elastic energy stored in an electromechanical resonant system could escape the system in the form of acoustic or electromagnetic waves (acoustic phonons or photons) or irreversibly transform to heat (thermal phonons) within the structure. In this chapter we briefly describe the major mechanisms for such energy loss (i.e., damping) processes. Given the energy loss through all damping mechanisms is known, the overall quality factor of a resonator can be found by summing up the dissipated energies [94]:

(12) $$Q_{total}={\left(\sum \frac{1}{Q_{i}}\right)}^{-1}$$

where $Q_{i}$ corresponds to damping from each potential loss mechanism.

### 5.1. Viscous Losses

Anytime a resonator operates inside a fluidic (gaseous or liquid) medium the resonator boundaries/surfaces continuously push against the surrounding molecules and transfer a portion of the resonator kinetic energy to the surrounding. This mode of energy loss is known as viscous loss or more particularly air damping (when the resonator operates in air). Viscous losses are a dominant source of loss in micromachined resonators as the surface to volume ratios become significantly larger at micro-scales [95]. The air damping is dependent on a variety of parameters including the resonator dimensions, the distance between the moving body of the resonator and the surrounding fixed surfaces (such as the electrodes in capacitive resonators or package walls), the frequency of operation, the resonance mode (i.e., how surfaces move relative to each other), and the gas pressure surrounding the resonator. Considering such complexity, one characteristic parameter that is commonly used to analyze the air damping is the Knudsen number (*K_n_*). In this context, *K_n_* is defined as the ratio of the fluid molecular mean free path to the separation between the resonator and the fixed surrounding structures. The *K_n_* > 1 range is of practical significance as the majority of vacuum packaged resonators operate under this condition. In this regime of operation, the interaction of air molecules could be ignored as the collision of the air molecules with the resonator plays a dominant role. There have been several attempts to model the air damping in this regime with reasonable success [96,97,98] but an accurate prediction of the loss appears to be yet out of reach.

**Figure 12:**
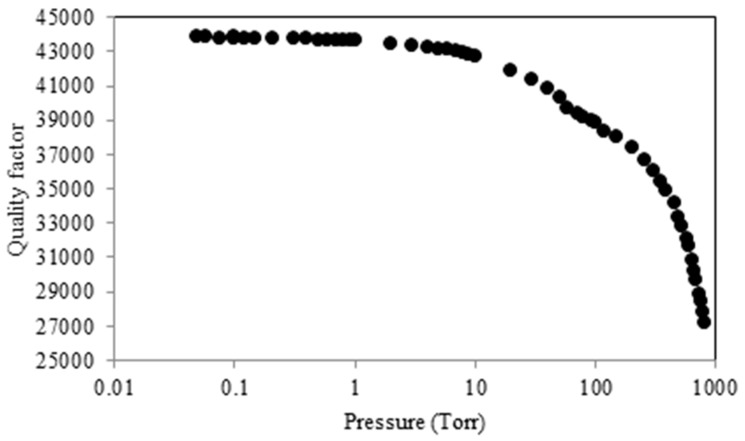
A typical measured Quality factor vs. pressure plot for micro-scale (MEMS) resonators.

In practice, the air damping is often avoided by packaging the resonators in partial vacuum. The *Q* vs. pressure graphs for micro-resonators typically follow a trend similar to what is shown in Figure 12. As seen in this figure, beyond a certain vacuum level, the effect of pressure (air damping) is negligible and other sources of loss dominate the effective quality factor. This is exactly the range targeted by the manufacturers as the sharp change of *Q* as a result of the variation in pressure could be detrimental to the performance and should be avoided.

### 5.2. Anchor Losses

**Figure 13:**
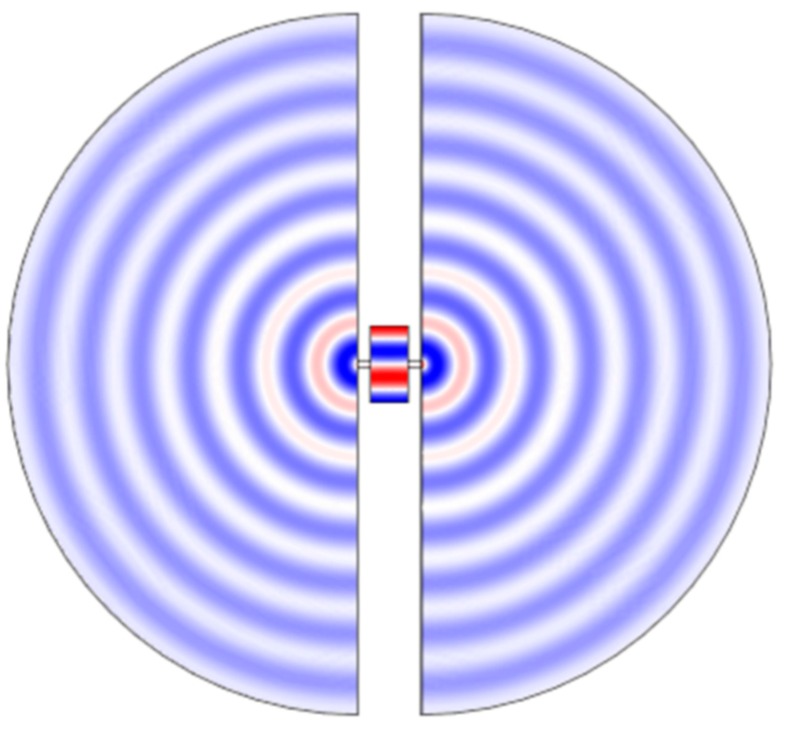
2-D model of a third harmonic lateral-extensional resonator. The strain is color-coded on the resonator and the substrate but the color intensity is not a true representation of the strain intensity as it is manipulated on the substrate region to enhance visibility.

Most resonators have to be suspended through mechanical connection(s) commonly known as anchors that attach the resonator to a supporting frame. At resonance the elastic waves trapped in the resonator can leak through these same connections and propagate to the frame causing loss of energy. This type of loss is often called anchor/support loss (Figure 13). From this definition it is perceived that anchor loss is strongly dependent on the location and the size of the anchor. Analytical prediction of anchor loss is complicated and is accomplished for a limited class of resonator (mainly beams) [99,100]. However, finite element models capable of capturing the anchor loss are developed in recent years and are finding increasing popularity among designers to suppress the anchor loss during the design phase [101,102].

A universal guideline for mitigation of anchor loss is to reduce the cross sectional dimensions of the anchor-to-resonator connection relative to the acoustic wave-length and to align the center of the anchors to the nodal points of the resonance mode where the particle displacement on the resonator body is minimum [18,103]. However, there are limitations in implementation of this guideline due to fabrication imperfections such as misalignment [63] and as the acoustic wavelength reduces beyond the smallest feature sizes practically feasible (for high frequency devices).

An alternative approach explored by researchers is to add features around the resonator that effectively reflect a portion of the radiated elastic energy back to the resonator. Such acoustic reflectors could simply be trenches etched into the substrate [104,105] or phononic crystal structures that are tuned to block a narrow band centered around the frequency trapped in the resonant cavity [106,107]. Phononic crystals can also be embedded in the design of the suspension tether [108,109].

### 5.3. Material Losses

Both viscous and anchor losses share the same characteristic in that the resonator elastic energy leaves the resonator for both mechanisms. In contrast there are a variety of mechanisms through which the elastic energy irreversibly turns into heat within the body of the resonator; hence categorized as material losses. The most fundamental and general approach to understanding such losses is through a quantum mechanical view. In quantum mechanical terms, the quanta of vibration energy are called phonons. Based on this definition, the elastic energy stored in a resonator is carried by phonons (elastic phonons). Similarly, heat energy, which is basically the random vibrations of particles, is embodied by phonons as well (thermal phonons). With this brief introduction, it can be envisioned that elastic phonons interact with other quantum mechanical particles inside the resonators including thermal phonons and electrons through a scattering process [110]. The end result of such intercalations is transformation of elastic energy to heat within the resonant body. The loss associated with the phonon-electron interaction is understandably not significant in dielectric and lightly-doped semiconducting material which are commonly used as the bulk of the micromachined resonant body.

In all resonators, depending on the relative values of the mean phonon scattering time ($\tau_{s}$), elastic vibration period ($\tau_{v}$), and the thermal transport time constant ($\tau_{\text{th}}$) a certain phonon-phonon interaction process could become dominant [111]. In flexural resonance modes of beams when τ_v_ ≈ $\tau_{\text{th}}$ (i.e., the average time it takes for phonons to transport between the local hot and cold spots is equal to the vibration period), elastic phonons efficiently interact with thermal phonons through a diffusion process. This process is classically described as thermoelastic damping (TED) [112]. TED in bulk mode resonators is insignificant and is practically absent in shear mode devices. A great body of effort exists on analytical and numerical modeling of TED [112,113,114,115,116]. Researchers have also attempted to reduce the effect of TED by engineering $\tau_{\text{th}}$ to be as far from $\tau_{v}$ as possible [117,118].

**Figure 14:**
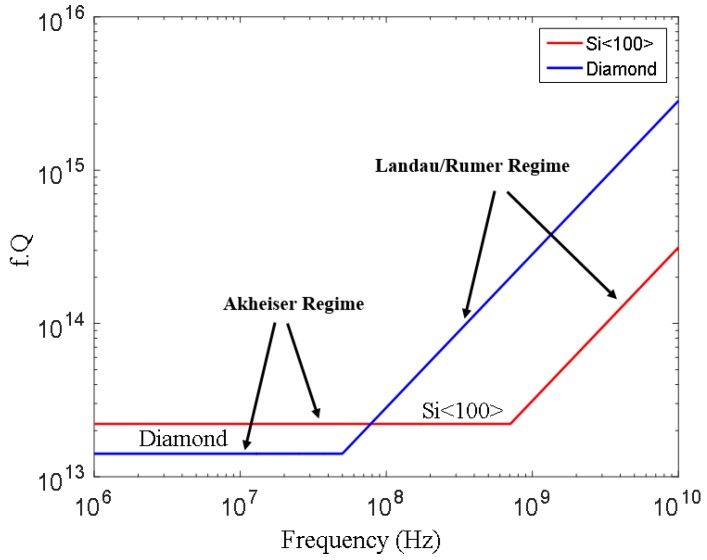
Limit of *f*.*Q* for a wide range of frequencies imposed by phonon scattering in diamond and <100> Silicon.

Apart from TED, in all resonators the periodic change of atomic spatial arrangement will disturb the equilibrium phonon distribution and as long as $\tau_{s}<\tau_{v}$ it results in a redistribution of phonons through phonon-phonon scattering. This process is known as Akheiser loss and sets a fundamental limit on the quality factor that could be achieved in a resonator depending on the material chosen for the resonator [110]. This loss is proportional to vibration frequency and becomes more significant at high frequencies (f>100 MHz for most relevant acoustic material). It should be noted that Akheiser loss is no longer effective for $\tau_{s}>\tau_{v}$ (very high frequencies) and the limit of quality factor could be relaxed at such frequencies [119]. This new regime of high frequency loss was first described by Landau and Rumer and the onset of this loss process (i.e., $\tau_{s}=\tau_{v}$) could greatly vary between different materials (Figure 14).

### 5.4. Other Damping Sources

There are several other pathways for the elastic energy to turn into heat including (but not limited to): ohmic losses due to electrical currents passing through resistive paths, dielectric losses [120] due to established electric fields across dielectric films, and surface losses [121] due to non-idealities associated with surface roughness and contaminations and electromagnetic radiation losses due to variation of electric fields. Ohmic and dielectric losses are relatively straight forward to predict and manage, however, the physics of surface losses are rather complicated. Regardless of such complexities it is been practically shown that the surface losses could be minimized through vacuum annealing and avoiding large surface to volume ratios [122].

## 6. Transduction Mechanisms

In most applications, micromachined resonators are interfaced with electronic circuits. Therefore, the mechanical vibration in the resonator should be excited and sensed by an electrical signal (i.e., change in a voltage or a current). The choice of mechanism through which the electrical energy is reciprocally converted to elastic energy (i.e., mechanical vibration) plays a critical role in the overall performance of a product that contains the resonator. Factors associated with the transduction mechanism such as efficiency of the energy conversion (i.e., coupling coefficient), implementation simplicity, and power consumption should be carefully considered and analyzed. In this chapter the most commonly used transduction mechanisms are briefly discussed and their main properties are highlighted.

### 6.1. Capacitive

A voltage applied between two conducting plates separated by an insulating medium generates a force that could move the plates given one is free to move. Reversely, change of capacitance as a result of movement induces an electrical current given a constant voltage is applied to the conducting plates of the capacitor. This is the original mechanism exploited in capacitive resonators. Capacitive transducers are relatively easy to implement as there is no special requirement on the choice of material except for high electrical conductivity of the electrode plates. With this, it is no surprise that the first published micromachined mechanical resonators operated based on capacitive transduction [2] and it continues to be a very common choice for implementation of such resonators.

**Figure 15:**
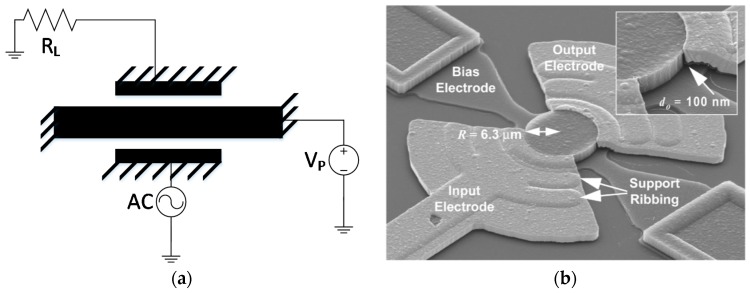
The schematic representation of a two-port capacitive beam resonator (**a**) and the scanning electron micrograph of a capacitive polysilicon disk resonator (**b**) [62]. ^©^ 2005 IEEE. Reprinted with permission from High-Q UHF micromechanical radial-contour mode disk resonators by J. R. Clark in *J. Microelectromech. Syst.*, 2005.

The essential components in a capacitive beam resonator are schematically shown in Figure 15 for a two-port configuration. The alternating input electrical signal in this diagram is applied through a fixed electrode on one side to excite the mechanical vibration and on the other side the mechanical movement is converted back to an electrical current in a symmetric design. A DC voltage labeled *V_p_* (polarization or bias voltage) is connected to the resonator body to establish the required initial electric field.

As seen in the above schematic diagram, the resonant body usually constitutes one of the electrodes in a capacitive resonator. Therefore, the material used for the resonant body is required to be highly conductive. In the past, doped silicon/polysilicon [13,123] and doped polycrystalline diamond [124] have been the most common choices of material for a capacitive micro-resonator. Silicon and polysilicon are the most natural choice considering that the micro-fabrication industry is mainly developed around processing silicon-based material. Silicon is coincidentally an excellent choice of material for its excellent mechanical properties including, low loss and exceptional mechanical/chemical stability (i.e., negligible change of properties over time). Some of the highest *f.Q* products measured from MEMS resonators are reported for capacitive resonators as the resonant body could be fabricated from a single material which eliminates any interfacial loss existing in multi-layer resonators [125,126].

It can be shown that the electromechanical coupling factor for a capacitive resonator, defined as the ratio of the output mechanical force over the input electrical voltage, is derived from [94]:

(13) $$\eta =V_{p}\frac{dC}{dx}$$

where *V_p_* is the polarization voltage, *C* is the transducer capacitance, and *x* is the resonator displacement. From this equation one could conclude a number of basic properties of capacitive transduction. First, it is observed that the electromechanical coupling at microscale is a very small number (e.g., $\eta ≅10^{-7}$ for *V_p_* = 10 V, Capacitive Area = 10^−9^ m^2^, Capacitive Gap = 10^−6^ m, and assuming a parallel plate displacement in vacuum). This implies that capacitive transduction is not inherently an efficient energy coupling mechanism. Secondly, it is observed that the electromechanical coupling could be improved by increasing the polarization voltage and increasing the rate of capacitance change with respect to displacement which is proportional to the capacitive area and inversely proportional to the second power of capacitive gap. Several approaches have been explored by designers to improve the energy coupling. These range from simply increasing the capacitive area [58] or reducing the gap size to extremely small values [127]. Both of these approaches encounter limitations when the frequency of operation is pushed beyond 100’s of MHz as the acoustic wavelength is excessively reduced and so should the resonator’s critical dimensions. The alternative solution for improving the coupling at higher frequency is by coupling a large number of resonators to each other [79]. This approach although very effective adds to the fabrication complexity and may lower the fabrication yield.

A different approach to implement capacitive transduction is to use a solid dielectric to fill the gap between the electrode and the resonant body [128]. This class of resonators is relatively simple to fabricate as the deposition and removal of sacrificial material within the capacitive gaps which is a major source of failure is completely eliminated. Solid dielectric gap resonators have been demonstrated with reasonably high-Q at frequencies well beyond 1 GHz [129]. However, the use of a dielectric material with large permittivity will directly contribute to a large feedthrough capacitance that masks the resonance signal and will complicate the usage of such resonators in applications such as oscillators.

### 6.2. Piezoelectric

Piezoelectric resonators operate based on the direct conversion of electric polarization to mechanical stress (and vice versa) in a certain class of crystalline materials known as piezoelectric materials [130]. Piezoelectric resonators such as Quartz have been in use for many decades and are still the most prevalent technology in electronic applications. The main attractions of the piezoelectric transduction are the self-generating nature (there is no need for an electrical bias or power consumption) and the relatively large coupling coefficient indicative of efficient reciprocal conversion of electrical and mechanical energy. The main technical difficulty in working with piezoelectric material at micro-scale is their incorporation into mainstream microelectronics fabrication processes.

Single crystalline piezoelectric material such as quartz and lithium niobate cannot be simply grown on a silicon surface in the form of thin functional film. Therefore, alternative deposition techniques for deposition of properly oriented polycrystalline piezoelectric material should have been developed before piezoelectric micromachined resonators could be considered relevant. Moreover, many piezoelectric materials contain metals with high diffusivity or toxicity (e.g., Zinc oxide (ZnO) and Lead Zirconate Titanate or (PZT)) which cannot be tolerated in microfabrication facilities.

Some of the earliest instances of micromachined resonators were fabricated based on RF sputtered ZnO thin-film deposited on silicon substrate [131]. However, ZnO is a chemically-unstable material and resonators fabricated of ZnO have not been successfully commercialized. It was until the development of RF sputtered piezoelectric Aluminium nitride (AlN) [132] that the thin-film piezoelectric material slowly gained acceptance in microfabrication industry. In contrast to ZnO, AlN is a chemically stable material with excellent acoustic properties such as large stiffness and low loss. More importantly aluminum, the only metallic ingredient in AlN, is commonly used for metallization in microelectronics.

**Figure 16:**
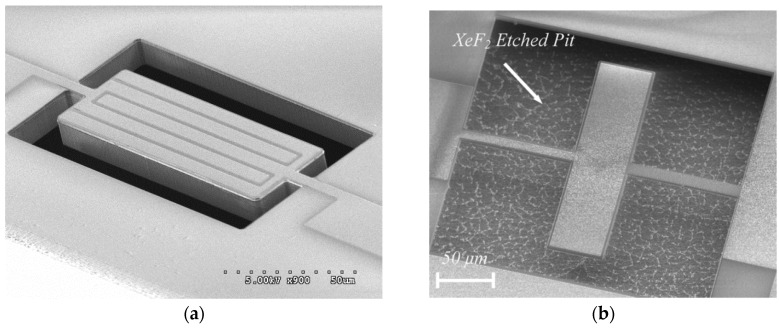
Scanning electron micrograph (SEM) of an AlN-on-Silicon fifth-order lateral-extensional mode (**a**) and an AlN contour-mode rectangular resonator (**b**) [135]. ^©^ 2007 IEEE. Reprinted with permission from Enhanced Power Handling and Quality Factor in Thin-Film Piezoelectric-on-Substrate Resonators by R. Abdolvand in Proceedings of the IEEE Ultrasonics Symposium, 2007.

Thin-film piezoelectric micro-resonators could be divided into two main categories. The first category is the devices that use the thin-film piezoelectric layer mainly as a transducer to generate/sense the acoustic waves in a second substrate material [131,133,134]. Such devices are sometimes referred to as thin-film piezoelectric-on-substrate (TPoS) resonators (Figure 16) and can significantly benefit from the proper choice of the substrate material to improve certain features of the resonator characteristic such as the quality factor and linearity [135]. In addition to common choices of substrate material such as Silicon, polycrystalline Diamond has been demonstrated to be an excellent choice for high frequency applications [136,137]. The combined high-Q and low motional resistance offered by TPoS devices enabled the demonstration of some of the best oscillator performances achieved from MEMS resonators [138,139]. The trade-off in using a substrate under the piezoelectric layer in a TPoS resonator is the compromised coupling factor.

The second class of micromachined piezoelectric resonators utilizes the piezoelectric film both as the transducer and the acoustic media. This category conceptually include the very mature thickness-mode film bulk acoustic resonator (FBAR) technology [66] as well as the more recently developed contour-mode devices [65,140]. Devices of this category offer low-motional resistances at very high frequencies that are unmatched by any other MEMS resonator technology and are specifically useful for filter applications [141,142]. However, the quality factor of such resonators is inferior to the capacitive resonator and TPoS resonators especially at lower frequencies (f<500 MHz).

### 6.3. Thermal/Piezoresistive

Unlike capacitive and piezoelectric transducers, there are other transduction mechanisms that could only be used to either excite the vibration or sense the vibration (i.e., one-way transduction). For example, thermal actuators could only be used for excitation of vibration and piezoresistive elements could only be used to sense the change in the resistance as the resonator vibrates. Despite such relative deficiency both thermal and piezoresistive transducers are very attractive for their ease of implementation. All that is required in both cases is a conductive material through which an electrical current is passed to either generate heat (in the case of the thermal actuation) or to measure resistance (in the case of the piezoresistive sensing).

In a thermally-actuated resonator, an alternating current is passed through the resistive heating elements to generate a dynamic heating power. This varying power will result in a dynamic temperature distribution (thermal wave) in the resonant structure which is the source of the desired actuation force. Once the frequency of the thermal wave matches the mechanical resonance frequency of the structure the mechanical vibration is efficiently excited [94]. Thermal actuation is specially desired for applications in which a large force is required for excitation of the vibration in liquid medium [143]. The efficiency of the thermal transduction (force to heat ratio) is dependent on the thermal time constant associated with the structure. Generally speaking, the equivalent model of a heat generator with a heat transfer path can be simplified to an $R_{TH}C_{TH}$ circuit where $R_{TH}$ is the thermal resistance associated with the heat transfer and $C_{\text{TH}}$ is the thermal capacitance. In other words, the temperature (i.e., force) generated by an input alternating power reduces for higher frequencies. This fundamental behavior has led to the traditional belief that thermal actuators are “slow” and can only be used for low frequency applications. However, there is a growing body of evidence pointing to the contrary. Based on some recent and original work on this topic, the thermal actuation can be used for very high frequency applications [56]. It could be proved that the thermal time constant for a specific resonant structure scales much faster (it has second order dependency) than the resonant frequency (liner dependency) as the dimensions of the structure reduce [144]. In other words, the temperature of a structure follows the input power much faster (less lag) as the dimensions are scaled down. A fundamental limitation associated with the thermal actuation that continues to impede its spread is the required power consumption to generate considerable vibration amplitude especially at higher frequencies where the structure is stiffer and the amplitude of the alternating temperature is lower for the same input power.

**Figure 17:**
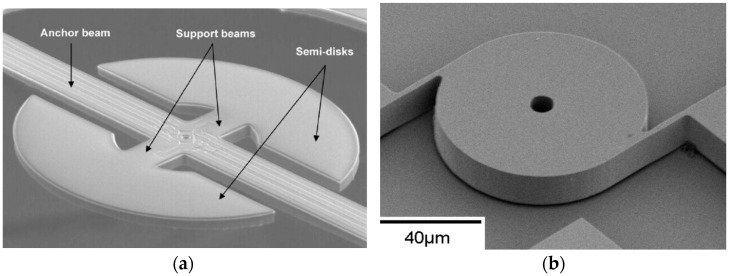
SEM of piezoeresistively-sensed thermally-actuated resonator: a rotational disk resonator with boron-doped piezoresistive readout element (**a**) and a solid single crystalline silicon disk resonator with combined heater/piezoeresistive silicon beams (**b**) [147]. ^©^ 2010 IEEE. Reprinted with permission from Rotational mode disk resonators for high-Q operation in liquid by A. Rahafrooz in Proceedings of the 2010 IEEE Sensors, 2010.

In thermal resonators the mechanical vibration is commonly sensed through piezoresistivity which is the change of resistivity in response to stress. In the most general form the piezoresistivity in material is characterized by a 6 × 6 matrix of piezoresistive coefficients. Semiconductor materials such as doped single crystalline silicon possess exceptionally large piezoresistive coefficients [145] which enables an efficient sensing vehicle. Piezoresistive elements could be formed either by deposition and patterning of a thin film or selectively doping the surface of the silicon substrate [146] separate from the heating resistor, or alternatively be formed from the bulk of silicon [147] (Figure 17). The latter is an attractive approach as the same heating element could be used as the piezoresistive sensing element simplifying the device interface (a two-terminal interface as opposed to four-terminal).

Piezoeresistive sensing has been also coupled with other actuation mechanism such as capacitive to improve the effective electromechanical coupling [148] as piezoresistive coupling can be enhanced through increasing the readout current. Piezoresistivity is also the transduction of choice for extremely small scales [149,150,151] as other transducers lose efficiency while piezoresistivity enhances [152]. Piezoresistivity is also the most compatible transduction with mainstream CMOS fabrication as minimum alteration to the process is required [153].

### 6.4. Other Transduction Mechanisms

Beyond the most common transduction schemes discussed above, several other energy conversion processes could be utilized for specific applications. For example, electromagnetic Lorentz forces have been successfully utilized to excite vibration in micromachined resonator [154,155]. Optical sensing is another mechanism that is relatively popular amongst researchers for detection of the structural vibrations as the sensing apparatus is completely independent of the resonant structure and can be used for a wide variety of resonators [156].

## 7. Fabrication

### 7.1. Narrow Gaps

As was previously described, the electromechanical behavior of a resonator oscillating in the linear regime can be modeled using an inductor-resistor-capacitance (LRC) series resonant circuit representation. It has also been pointed out earlier that the motional resistance scales inversely with the fourth order of the capacitive transducer gap for resonators that are actuated and sensed by capacitive transduction. Given the importance of narrowing the transduction gap in order to reduce motional resistance, methods for fabricating narrow gaps at the scale of sub-microns have been reported for polysilicon-based and silicon-on-insulator-based fabrication processes.

**Figure 18:**
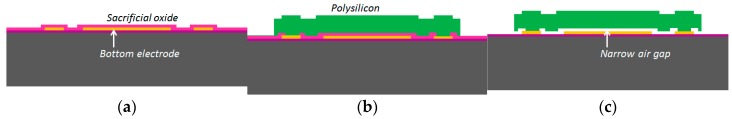
Fabrication of vertical narrow gaps in a polysilicon-based process: (**a**) Growth of sacrificial oxide; (**b**) Growth and patterning of polysilicon structural layer after patterning of sacrificial oxide; (**c**) Release of polysilicon structure of removal of sacrificial oxide.

In the case of polysilicon beams that were designed to vibrate out of plane that require a vertical capacitive gap, the gap size is defined by the thickness of a silicon oxide layer typically grown by low pressure chemical vapor deposition (LPCVD) that acts as a sacrificial layer [18,86]. The oxide layer can be made as thin as 130 nm. The thin sacrificial oxide layer is patterned (etched away where the anchoring region are located), followed by deposition of the polysilicon structural layer. After the polysilicon layer is patterned to form the beam structures, the oxide layer is etched away by buffered hydrofluoric acid (HF) to leave behind a thin air gap between the beam and the drive electrode. The process flow for fabricating vertical narrow gaps in a polysilicon-based process is illustrated in Figure 18.

**Figure 19:**
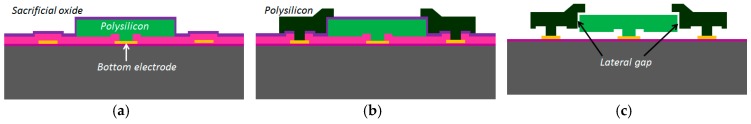
Fabrication of lateral narrow gaps in a polysilicon-based process: (**a**) Conformal growth of sacrificial oxide to define lateral gaps; (**b**) Growth and patterning of polysilicon electrodes; (**c**) Release of polysilicon structure after removal of sacrificial oxide.

While the process flow described in Figure 18 is suitable for realizing out-of-plane vibrating beams that require vertical capacitive gaps, the process has to be modified to order to realize lateral capacitive gaps for the case of laterally vibrating bulk mode resonators. The modification in the process requires extra steps to define the narrow lateral gap as illustrated in Figure 19. As can be seen from Figure 19, after the polysilicon structure layer that defines the resonator has been patterned, a conformal side wall high temperature oxide (HTO) film coats the whole structure (including side walls) by LPCVD. The thickness of the conformal side wall HTO film defines the gap separation of the lateral electrodes. This is followed by LPCVD low-stress polysilicon to form the side electrodes, which is subsequently patterned to define the structure of the electrodes. The structure is finally released by HF etch, that also removes the side wall HTO to leave behind a narrow air gap as thin as 30 nm [62,63].

**Figure 20:**
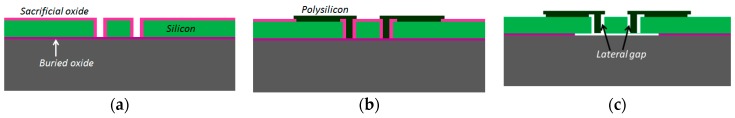
Fabrication of narrow gaps in a silicon-on-insulator-based process using polysilicon refilling of the gaps etched into the silicon device layer by Deep Reactive Ion Etching: (**a**) Conformal growth of sacrificial oxide to define lateral gaps; (**b**) Growth, etch back and patterning of polysilicon electrodes; (**c**) Release of silicon structure after removal of sacrificial oxide.

Compared to micromachining MEMS resonators with polysilicon, fabricating MEMS resonators based on silicon-on-insulator (SOI) wafers offers the advantage of having thicker structural layers available. The challenge for realizing thick structures in polysilicon lies in keeping the film stress low. In an SOI process, the resonator structure is almost always defined by the silicon device layer, which comes available in a variety of thicknesses even up to 100 µm. In the case of laterally vibrating resonators, thicker structures are desirable for the reducing motional resistance. Narrow gaps are still needed in SOI-based resonators in order to improve electromechanical coupling. In order to realize narrow lateral capacitive gaps in an SOI process, the approach of using conformal side wall oxide to define the gaps needs to be modified. As illustrated in Figure 20, the key difference of fabricating narrow gaps in single-crystal silicon is instead of growing a polysilicon electrode, the deposited polysilicon is used as a filling material. The lateral gap is first formed by etching vertically through the single-crystal silicon by deep reactive ion etch (DRIE). Although DRIE allows for high aspect ratio trench structures, the desired narrow gaps demand greater precision not practical for DRIE. Hence the initial gap defined by DRIE is narrowed by first depositing a conformal LPCVD HTO followed by refilling the HTO-coated trenches with polysilicon. Once again, the electrode gap is defined by the thickness of the conformal side wall oxide. When the sacrificial oxide is removed by HF etch, a narrow submicron air gap is formed [123,157,158].

Some research groups have explored the benefits of defining the gap with a solid dielectric, substituting the silicon dioxide film with a different film that has a higher dielectric constant, such as silicon nitride. The process flow is the same to what is depicted in Figure 20, except that the nitride is not etched away. The benefit of this approach is that it allows thinner gaps to be realized, while additionally increasing the capacitance by means of the high dielectric constant of the film [159,160]. It has been found that the best location on the resonator to place the solid gaps is at the points of minimum displacement [128].

Others have also shown notable reduction in motional resistance by using a partially filled gap whereby a part of the air gap between the structural side walls is filled by a high-K dielectric. The high-K dielectric film thus increases the dielectric constant of the gap electrode overall while still leaving a thin air gap [160]. This approach aims to deliver high quality factor by keeping an air gap while improving the coupling efficiency through gap reduction and increasing the overall dielectric constant of the gap.

### 7.2. Piezoelectric Layers

As mentioned in the previous section, fabrication of narrow gaps is essential to improving transduction in the case of capacitive resonators. This brings along additional complexity to the fabrication process. Piezoelectric transduction offers an alternative to achieve reduced motional resistance while leveraging on developments in the growth of piezoelectric thin films owing to the maturity and success of thin film bulk acoustic resonators (FBARs). Among piezoelectric thin films that have been applied to MEMS resonators, AlN [65,161,162,163], ZnO [133], and PZT [164] are the most common ones reported in the literature. On this note, AlN has become highly popular due to its compatibility with existing fabrication technology for manufacturing integrated circuits. The cross section of AlN resonators generally comprises a thin AlN film that is sandwiched by bottom and top metal electrodes. The bottom electrode acts as a ground, and input AC voltage is applied to the top electrodes. This results in an electric field that is dropped across the thickness of the AlN structure. These AlN resonators are designed to vibrate primarily in the lateral modes, of which the frequency is defined by the lateral features of the resonators (as mentioned in Section 6.2 previously). Hence although the cross-sectional topology of the AlN MEMS resonator is similar to an FBAR, the modes of interest are different as the FBAR. The FBAR is designed to vibrate across the thickness of the film, which thus defines the resonant frequency. The motivation behind AlN MEMS resonators is to realize integrated resonators that are characterized by low motional resistance and resonant frequencies that can be lithographically defined towards a multiple-frequency on a single chip solution. As such, given that the electric field is applied across the thickness and the intended mode of vibration lies within the fabrication plane, the vibration modes are excited and detected through the d_31_ piezoelectric coefficient. FBARs in contrast are transduced through the d_33_ coefficient. Given that the d_31_ coefficient is typically lower than the d_33_ coefficient, the coupling efficiency of laterally-vibrating resonators is thus generally lower than an FBAR for the same piezoelectric material.

Apart from realizing a piezoelectric resonator with only the piezoelectric film to define the structural layer, some research groups have implemented resonators comprising a thin piezoelectric film on a thick substrate layer. In this case, the structural layer is defined mainly by the substrate material, examples of which include single-crystal silicon [59,165,166,167,168], silicon carbide [169,170,171], and diamond [172,173].

Pursuant to reaching higher coupling coefficients towards realizing low insertion filters based on laterally vibrating resonators, some groups have turned to materials with higher piezoelectric coupling coefficients such as Lithium Niobate (LiNbO_3_). The process differs from the above as device is fabricated from the wafer itself as the material cannot be deposited as a thin film. MEMS resonators fabricated from LiNbO_3_ have been shown to exhibit much lower insertion losses compared to AlN resonators [174,175,176,177].

### 7.3. CMOS MEMS

While the above processes involving silicon processing and deposition of AlN films are compatible with CMOS fabrication technology, some groups have been explored fabricating MEMS resonators based on standard CMOS technology. The key advantage of this approach, referred to CMOS MEMS, is monolithic integration of the MEMS structure with the interface electronics. Most demonstrations of CMOS MEMS resonators have been reported for either 0.35 µm or 0.18 µm technologies. There are several ways in which a MEMS structure can be realized from the layers of polysilicon, interlayer dielectrics, and metals included in a standard CMOS process.

One approach is to use the polysilicon layers to define the MEMS resonator and electrodes. In the case of [178,179,180,181,182] where there are two polysilicon levels, one polysilicon level was used for the resonator while the other polysilicon level was used for electrodes. In this case, the oxide layer between the two polysilicon levels defines the lateral gap between the side electrode and beam resonator, allowing gaps as narrow as 40 nm to be realized. As such, the oxide layer here serves as a sacrificial layer. The thickness of the resonator is defined by the polysilicon thickness, which is typically a few 100 nm and therefore much thinner than the polysilicon resonators described in Section 6.1 previously. This in turn places a limit on the transduction efficiency.

The other approach found from the literature is to use the top metal layer to define both the MEMS resonator and the electrodes [183,184]. In this case, the minimum achievable gap between the electrode and the MEMS structure is determined by the technology layout rules (i.e., the minimum gap between two features defined in the same metal level allowed in the process). In this case, as the MEMS resonator is defined in the metal layer, the thickness of the MEMS resonator will be determined by the thickness of the metal layer, which can be almost 1 µm. Compared the previously mentioned approach of using a spacer, realizing the MEMS resonator with the top metal layer allows a thicker structure but with the drawback of a wider capacitive gap.

The above two approaches define the resonator using the conductive layers that are available in the CMOS technology stack, of which only one layer is used. The last approach in contrast uses several of the layers that include both metals as well as interlayer oxide layers in order to realize a structure that is much thicker [185,186,187]. In this approach, the metal layers are physically and electrically connected to each other vertically through vias. These interconnected metal features are used to realize conducting sidewalls in both the beam resonator and the electrodes, embedded within an oxide matrix formed by the different layers of interlayer oxide. The other function of the interconnected metal features is to serve as vertical sacrificial features that extend through the entire thickness of the MEMS structure. These sacrificial metal features are exposed to the etching solution that selectively etches away the metal, while the metal sidewall features embedded in the oxide matrix are protected from the etchant. There is also the final option to release the structures from the underside by etching the exposed top side of the silicon substrate with XeF_2_. Out of plane vibration mode structures can also be realized using this approach and integrated with a MOS field effect transistor (FET) to implement a resonant gate FET [187] that has the advantage of incorporating intrinsic gain.

### 7.4. Packaging

Adequate packaging of resonators for the purpose of extending reliability is essential given that these devices are extremely sensitive to the external conditions. The package provides a barrier against exposure of the device to dust and moisture. In the particular case of capacitive resonators, the device needs to be sealed at moderate vacuum pressure levels in order to reduce viscous damping. There are two main approaches that have been used to seal the device at the wafer-level.

The first involves attaching a capping wafer onto the processed wafer with the resonator, which typically is either glass or silicon [188,189,190,191]. The capping wafer is typically processed by etching recesses into the wafer to create a cavity to accommodate the MEMS device to be encapsulated.

The other approach involves depositing an encapsulation material directly on the wafer processed with the MEMS device. Prior to the deposition of the encapsulation layer, a sacrificial layer is deposited over the processed MEMS device. The encapsulation layer could be a polymer [192] or combination of a metal and organic film [193,194,195] where the sacrificial layer is also an organic film. The benefit of using metal and organic films as the encapsulation layer is that the process can be done at low temperatures and thus compatible as a post-processing step. An alternative to implementing encapsulation as a post-processing step is to integrate packaging into the process for fabricating silicon resonators, using encapsulation materials such as polysilicon [195] or epitaxial silicon [196]. In this case, silicon oxide is used as the sacrificial layer that is deposited over the SOI wafer which has been processed with the resonator. The first encapsulation layer is then deposited and etched through to create vents. The resonator is released from the substrate and encapsulation layer through vapor HF etching, followed by sealing the wafer with a second encapsulation layer that seals off the vents.

## 8. Applications

### 8.1. Timing

For over a decade, MEMS resonator-based oscillators have moved towards commercialization for timing applications [8,10,12,197,198], mainly focusing on wired communications standards such as USB and on real-time clocks. The reason why MEMS oscillators are more slowly penetrating RF systems as frequency references is due to their fairly stringent phase noise requirements. These requirements stem from the synthesized carrier spectral purity specified by the majority of wireless standards. The close-in phase noise performance requirements are particularly challenging in wireless standards, as resonator non-linear behavior and somewhat lower-Q-factor than quartz usually degrades performance at close-in offsets to be as competitive for such applications. However, in serial communications, where clock-data recovery circuits filter close-in phase noise due to their feedback nature, close-in phase noise performance is relaxed, allowing MEMS resonator-based oscillators to penetrate these applications regardless of their somewhat lower close-in phase noise performance [15,199].

**Figure 21:**
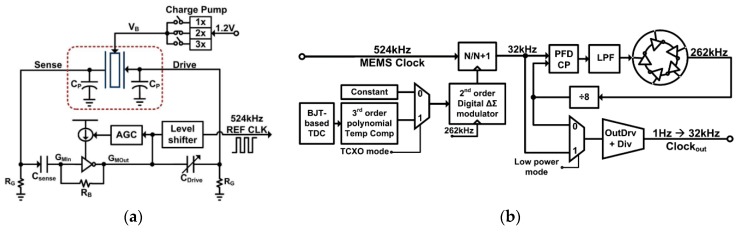
(**a**) A MEMS oscillator and (**b**) its compensation and frequency synthesis electronics [202]. ^©^ 2015 IEEE. Reprinted with permission from A 3 ppm 1.5 × 0.8 mm^2^ 1.0 μA 32.768 kHz MEMS-Based Oscillator by Zaliasl in *J. Solid-State Circuits*, 2015.

In addition to phase noise performance, an important requirement of timing applications is the frequency stability of the oscillator. Recently, temperature compensation algorithms or resonator fabrication techniques have allowed MEMS resonators to match the performance of quartz temperature compensated oscillators (i.e., TCXOs) with regards to temperature stability [199]. Real-time clocks, requiring oscillators operating at 32.768 kHz are of particular interest such as demonstrated in [200,201,202,203], where the resonator-based oscillator is interfaced with a phased-locked loop to synthetize the desired frequency output, and improve its temperature stability through the use of a temperature sensor and calibration data. In [203], a phase-locked loop is not used for this purpose in order to reduce power consumption, but a state machine determines the fractional division ratio of the oscillator output based on the output of a temperature sensor and calibration data. This method achieves an output frequency stability of ±10 ppm over 0 to 50 °C. In [202], a resonator is placed in a Pierce oscillator loop shown in Figure 21a [202]. Its 524 kHz output is fed to a dual-mode compensation circuit that can generate the 32 kHz required output, shown in Figure 21b [202]. In compensated mode, a modified fractional-N phase-locked loop can be activated to provide precise temperature compensation by modulating its output frequency based on the output of a temperature sensor. This allows to maintain the output frequency steady regardless of frequency drift due to temperature in the MEMS oscillator. In low-power mode, the phase-locked loop can be bypassed in order to generate an uncompensated output for applications that do not require compensation and which can benefit from the reduced power consumption. In low-power mode, the current consumption is of 0.6 μA (1.4 V supply), and when temperature compensated it is of 1.0 μA (1.4 V supply). The system achieves a ±100 ppm frequency stability over −40 to 85 °C in low power mode, and of ±3 ppm in temperature compensated mode. Note that the temperature compensation in this system requires calibration to allow for the most effective compensation of the resonator’s temperature characteristic.

Similar efforts have also been done using phase locked loops to generate MHz range output frequencies for systems targeting serial communications such as in [204] where a 5 MHz MEMS resonator-based oscillator is used as the frequency reference of a fractional-N phase-locked loop to generate output frequencies ranging from 1 MHz to 110 MHz with a ±30 ppm stability from −40 °C to 85 °C. Another design improves the temperature compensation to ±0.5 ppm accuracy from −40 °C to 85 °C and widens the output frequency range from 0.5 MHz to 220 MHz, consuming 3.97 mA from a 3.3 V supply [205].

Alternatively, some other temperature compensation techniques rely on dual resonator devices that have different temperature coefficients and are placed in a thermal feedback loop. These attain frequency stabilities of ±1 ppm over −20 °C to 80 °C in [206], and of ±4 ppm from −40 °C to 70 °C in [207], and interestingly do not require calibration, which is a significant advantage in order to reduce device cost. However, these techniques require heating of the resonators, and thus will consume higher amounts of current that are on the order of a few milliamps.

Considering wireless standards, in [208], while no temperature compensation loop is implemented, a fractional-N phase locked loop with a MEMS resonator-based oscillator running at 11.6 MHz is presented with an RF output frequency of 1.7–2 GHz and attempts at meeting the local RF oscillator wireless standards performance metrics of GSM, such that the reported phase noise attained is −122 dBc/Hz at a 600 kHz offset, and −137 dBc/Hz at a 3 MHz offset. Close-in phase noise performance precludes the system from meeting wireless phase noise standards (e.g., error vector magnitude), because of the relative low Q-factor of the resonator used.

### 8.2. MEMS Resonator-based Oscillators

As was previously discussed, the electrical model applicable to all resonators is a series-resonant RLC circuit with capacitive feedthrough causing parallel resonance as well. If resonators are operated as channel-select filters such as in [209], typically no interface electronics are required to achieve the filtering operation. However, if present as filters in radiofrequency (RF) systems such as receivers, they may be embedded into electronic circuits such as mixers in order to reduce the impact of their typically higher insertion losses on the overall noise figure of the systems [15,210,211].

Typically, MEMS resonators are interfaced with sustaining amplifiers that allow for their use in electronic oscillators that generate an electrical signal at the resonant frequency of the resonator such as demonstrated in [211]. When used in sensors, resonators will usually operate through some functionalized MEMS resonator that varies its frequency in response to sensed element such as in [212]. When used in timing circuits as precision clocks [200] or as RF carriers [10], MEMS resonators are used as the frequency reference element. Whether MEMS resonators are used in sensing or in timing applications, the sustaining amplifier needs to carefully be designed to consider the particularities of MEMS resonators in order to enable high quality oscillation [213,214,215,216].

MEMS resonators, while similar in function to quartz crystals, have properties that require specific interface circuitry designs. For instance, unlike quartz crystals that can have milliamp scale output motional currents, the output motional current of a MEMS resonator is typically in the nano-ampere range [217,218]. In addition, their insertion losses, in the case of electrostatically driven resonators, can represent motional resistances in the 1 kΩ–100 kΩ range [18,62,218], which is significantly higher than quartz crystals (i.e., 25 Ω–200 Ω).

Accordingly, the power handling ability of MEMS resonators is on the order of a few micro-Watts, and they will exhibit significant non-linearity beyond that drive level which can deteriorate performance or, if harnessed properly by the electronics, improve it [219,220,221,222,223,224]. In addition, the Q-factor of MEMS mechanical resonators is in the 10^4^ range, and is generally strongly inversely proportional to the resonant frequency. This is lower than the Q-factors achieved by AT-cut crystals, which are typically in the range of 10^4^–10^5^ over a wide range of frequencies. Furthermore, the resonant frequency temperature dependence of an uncompensated MEMS silicon resonator is much higher (−30 ppm/°C [197]) than that of an AT-cut crystal (±25 ppm from −40 to 85 °C). Mechanical or electronic temperature compensation is thus a must for MEMS resonators to match quartz temperature stability, a critical parameter in all timing applications.

Regardless of their disadvantages, typical resonance frequencies of MEMS resonator can vary from the kHz range to the GHz range [63,203,225], which is much higher than what is attained by quartz crystals (~100 MHz), and they can be fabricated at relatively low cost and in some cases can be integrated monolithically with the electronics [12], which are significant advantages.

Ultimately, a MEMS resonator exhibits a series and a parallel resonance, and a sustaining circuit is required to compensate its motional resistance and provide the suitable phase condition to allow for electronic oscillation. However, the higher motional resistance, non-linearity and in some cases high operating frequencies increase the design complexity of the sustaining electronics. In addition, the mechanical noise of the resonator is an important factor to consider when designing an integrated circuit and several works have attempted to model it [221,226,227,228]. This section focuses on the particularities of MEMS resonator-based oscillators, and discusses the circuitry that is interfaced with MEMS resonators in order to implement them.

#### 8.2.1. Operating Principles

Oscillators are commonly used in RF systems in voltage controlled oscillators (VCOs) for high frequency signal generation of carriers, or as low-frequency reference oscillators for PLLs. They can also be used standalone in timing circuits, such as in electronic watches. Resonators are well suited to creating such oscillators, because of their high-Q-factors and frequency filtering properties [6,7,8].

**Figure 22:**
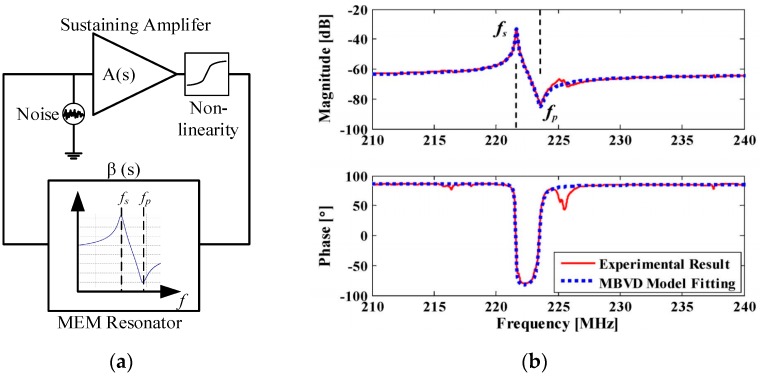
(**a**) Typical MEMS resonator-based oscillator loop; and (**b**) typical resonator transmission characteristic amplitude and phase [162]. ^©^ 2010 IEEE. Reprinted with permission from Multifrequency Pierce Oscillators Based on Piezoelectric AlN Contour-Mode MEMS Technology by Zuo in *J. Microelectromech. Syst.*, 2010.

The topology in Figure 22a illustrates the configuration of a typical MEMS resonator-based oscillator, with a resonator’s typical amplitude and phase frequency responses shown in Figure 22b [162]. In a *positive* feedback loop, a sustaining amplifier with a frequency dependent gain, A(*s*), an input-referred noise and a non-linear characteristic has its frequency response filtered by a MEMS resonator having a frequency dependent motional resistance and thus frequency response, β(*s*) [228]. At power up, the noise present in the positive feedback loop gets amplified and filtered by the resonator after multiple passes around the loop until the sustaining amplifier or the mechanical resonator limit the signal growth because of non-linearity. This reduces the loop gain A(*s*)β(*s*) such that in steady-state, the gain around the loop (i.e., loop gain) has an effective value of unity, and a sustained constant oscillation can be observed. Important aspects of the loop gain are that for this constructive positive feedback to occur, and to allow for an oscillation be sustained, the linear gain around the loop must be larger than unity, usually with some safety margin to allow for fast start-up and design margins (e.g., 1.5 times the minimal gain required), and the phase shift around the loop must allow for the noise waveform propagating around the loop to constructively grow. These oscillation conditions, first defined by Heinrich Georg Barkhausen, can be expressed as:

(14) |A(s)β(s)|>1

(15) ∠(A(s)β(s))=n360°, n=0,1,2…

As can be seen in the phase condition above, the phase shift around the loop must either be zero or a multiple of 360°. Typically, oscillators will either operate around a 0° phase shift or a 360° phase shift. In the former, the resonator’s series-resonance, when the resonator’s impedance is lowest, is used with a sustaining amplifier having sufficient bandwidth to add negligible phase shift to the loop, while in the latter, its parallel-resonance, when the resonator’s impedance is largest, is used with an amplifier providing 180° phase-shift around the loop. In that case, the rest of the phase shift is provided by electrical passive components, usually capacitors, such that the phase shift at a frequency between the series and parallel resonances of the resonators is of 180°, yielding the total required 360°. Typically, series resonance provides more accurate oscillation frequency as it does not depend on electrical components that may be inaccurate in order to attain additional phase shift, however, designing an amplifier with negligible phase shift can be a challenge [216,229,230]. Provided that the amplifier has enough gain to offset the loss of the resonator at resonance, and that its bandwidth is wide enough to contribute negligible phase shift to the loop, the circuit will oscillate at the series-resonant frequency of the resonator [230], otherwise, an offset in frequency will occur and the amplifier will have to provide more gain to overcome the additional losses of the resonator at a frequency offset from the series resonance. For parallel resonant circuits, a negative gain (i.e., a 180° phase shift) amplifier can also be used with additional phase shift, such as in Pierce oscillators [163,215].

#### 8.2.2. Phase Noise

**Figure 23:**
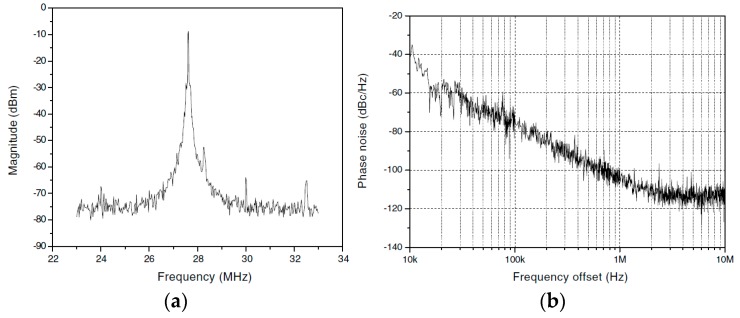
A 27 MHz MEMS resonator oscillator (**a**) output spectrum and (**b**) phase noise plot [232]. ^©^ IOP Publishing. Reproduced with permission. All rights reserved.

Because of the bandpass nature of the resonator, and the noise-shaping of the electronic amplifier noise caused by the feedback loop, the spectral density of the output is a single tone that has its frequency purity compromised by a “skirt” around it [231]. This is shown in Figure 23a [232], where the spectrum from a 27 MHz MEMS resonator oscillator is shown. The corresponding phase noise plot shown in Figure 23b [232], where the noise power relative to the oscillation power is plotted against offset frequencies form the oscillation frequency. In the time domain, phase noise can also be transposed to the time domain as jitter in the phase of the output signal. In timing applications, jitter performance is more often quoted (e.g., in ps) instead of phase noise performance (e.g., in dBc/Hz at a given frequency offset), but both metrics and inherently related [233].

**Figure 24:**
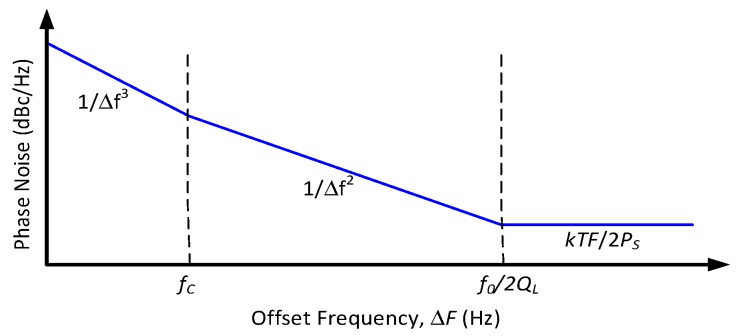
Typical phase noise power spectral density (single sided).

The Leeson phase noise model is a linear model that gives and expression for the phase noise in an oscillator. Leeson’s equation is given by [231]:

(16) $${ℒ}_{\varphi }\left(\Delta f\right)=10\;\text{log}\left[\frac{kTF}{2P_{S}}\left(1+{\left(\frac{f_{0}}{2Q_{L}\Delta f}\right)}^{2}\right)\left(1+\frac{f_{C}}{\Delta f}\right)\right]\Delta \text{dBc}/\text{Hz}$$

where *k* is the Boltzmann constant, *T* is the operating temperature, *F* is the effective excess noise factor, mainly caused by the sustaining amplifier, *P_S_* is the signal power at the input of the amplifier, *f_0_* is the oscillation frequency of the oscillator, Δ*f* is the offset frequency at which the phase noise is measured, *Q_L_* is the loaded quality factor of the resonator, and *f_C_* is the corner offset frequency at which the phase noise starts to increase at a rate of 30 dB per decade. The conceptual power spectral density of the phase noise shown in Figure 24 outlines that at far away offsets from the oscillation frequency, phase noise becomes white in spectrum, but within the half-bandwidth of the resonator (i.e., *f*_0_/2*Q_L_*), the phase noise increases by 20 dB per decade until it reaches a point where it increases by 30 dB per decade below *f*_C_. Leeson’s model is somewhat empirical as *F* and *f*_C_ are often obtained by measurements, since phase noise is often significantly affected by nonlinearities and time variance of the phase noise mechanisms in oscillators.

**Figure 25:**
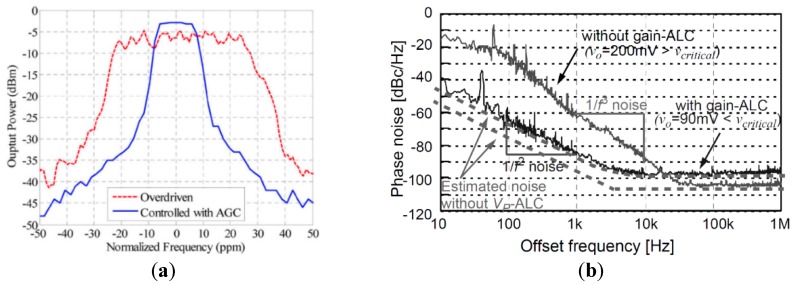
(**a**) The effect of automatic gain control due to the nonlinearity of the resonator on time domain frequency stability of a MEMS oscillator [208]. ^©^ 2009 IEEE. Reprinted with permission from A Highly Integrated 1.8 GHz Frequency Synthesizer Based on a MEMS Resonator by Nabki in *J. Solid-State Circuits*, 2009; and (**b**) the effect of automatic gain (level) control on the phase noise performance of a MEMS oscillator [237]. ^©^ 2003 IEEE. Reprinted with permission from Influence of automatic level control on micromechanical resonator oscillator phase noise. by Lee in Proceedings of the IEEE International Frequency Control Symposium, 2003.

More elaborate phase noise models exists such as that in [234], where a time-variant phase noise model is proposed. However, while not predictive in nature, the Leeson model can still provide important insights with regards to phase noise performance. Notably, a higher resonator quality factor and oscillation power will improve phase noise performance by tightening the oscillator’s output spectrum, and reducing excess noise stemming from electronics will improve the phase noise floor. It is also of interest to reduce the loading of the quality factor of the resonator by ensuring appropriate input and output resistances of the sustaining amplifier [235]. The 1/*f*^3^ corner frequency is often attributed to 1/*f* noise present in the electronics, however it is often different in phase noise plots, notably because of the time varying nature of the phase noise mechanism [234]. Degradation of phase noise due to noise folding can also impact oscillator performance [236]. In addition, complexities can arise because of nonlinearity [221] or the specificities of MEMS resonators can deteriorate phase noise. For instance, mechanical noise can impact performance depending on the relative noise performance of the sustaining amplifier [225]. Moreover, resonant non-linear behavior can also cause close-in offset noise degradation and a higher than 30 dB phase noise slope [222,226,227]. This degradation of phase-noise, notably at low frequency offsets has pushed most MEMS oscillator designs to employ automatic gain control in the sustaining amplifier in order to reduce the non-linear behavior of the MEMS resonator, and ensure optimal close-in offset performance [209,231,237,238]. The influence of automatic gain control on the oscillator phase noise is shown with regards to time-domain frequency stability in Figure 25a [208] and to phase noise performance in Figure 25b [237], outlining the significant degradation in oscillator performance when no automatic gain control is used to limit the oscillation amplitude. Notably, some resonator models have been proposed to enhance the typical RLC model which does not allow for circuit design that takes into account resonator nonlinearity (e.g., [223,228,239]), and other works have also leveraged the non-linearity of resonators to enhance phase noise performance by designing the oscillator to take advantage of the Duffing behavior of the resonator (e.g., [139,221]).

Ultimately, the limited power handling capabilities of MEMS resonators compared to quartz crystals restricts phase-noise performance improvement through the increase of the oscillation amplitude, but the high-Q they provide allows competitive phase noise performance if proper gain control measures are taken to mitigate resonator nonlinearity. This is particularly true of electrostatic resonators which are inherently more non-linear due to their actuation mechanism [83]. However, piezoelectric resonators are also prone to non-linear behavior [220]. Overall, the output phase noise of a MEMS resonator-based oscillator thus depends on several factors:

- The higher the Q-factor of the resonator, the lower the phase noise in the MEMS oscillator because of the enhanced noise filtering.
- The higher the power handling capability of the resonator, the lower the phase noise of the MEMS oscillator because of the increased sustainable amplitude of oscillation.
- The higher the motional resistance, the higher the phase noise of the MEMS oscillator because of the higher gain sustaining amplifier is required, usually required more active devices that generate noise.
- The lower the electronic noise of the sustaining amplifier, the better the phase noise because of the shaping of this noise that causes a significant portion of the overall phase noise.

#### 8.2.3. Temperature Compensation

A significant issue for MEMS oscillators is their stability with changes in the ambient temperature. As was previously mentioned, the resonant frequency temperature dependence of an uncompensated MEMS silicon resonator is much higher than that of an AT-cut crystal (i.e., ~50 times worse). Many strategies can be adopted to reduce this sensitivity. Electrostatic tuning can be used to change the bias of the resonator in response to temperature changes [240,241,242]. Thermal stress compensation can also be used by creating composite resonating structures that feature materials such as silicon oxide that can compensate silicon’s temperature variability [242]. Direct thermal tuning can also be used to control the resonator’s frequency and improve its frequency stability, similarly to oven controlled crystals [243,244,245]. Other approaches involve the use of phase-locked loops in arrangements that can include mismatched temperature coefficient resonators that result in a temperature stable operating point (e.g., [207,208]) or that can include temperature to digital converters that control the output frequency of the loop to compensate the resonator temperature variance (e.g., [203,206,207,246,247]). More recently, the use of electronics for compensation has precluded MEMS oscillators from operating at power budgets that rival that of quartz oscillators. However, mechanically temperature compensated resonators using doped silicon or silicon oxide [243,248,249] are a current interest for commercial applications as they can preclude the need for heating or electronic compensation, allowing for lower power consumption and better phase noise performance due to the reduced complexity of the control electronics.

#### 8.2.4. Sustaining Amplifiers

As was previously discussed, MEMS resonators exhibit very high motional resistances compared to that of quartz crystals—typically in the order of several tens of kilo-Ohms for electrostatically actuated resonators, and a few kilo-Ohms for piezoelectrically actuated resonators. Accordingly, in order to operate at the series-resonance of the resonator, the motional current outputted by the resonator device needs to be amplified by a trans-impedance amplifier (TIA) having the following characteristics [215]:

- a high gain to offset the resonator losses (i.e., at least 1.5 times the resonator’s motional resistance);
- a bandwidth which is an order of magnitude larger than the resonator’s frequency to ensure a small phase shift around the feedback loop;
- low input and output impedances to avoid loading the resonator’s Q-factor;
- an automatic gain control capability to prevent large oscillations from exerting the resonator’s nonlinearities.

**Figure 26:**
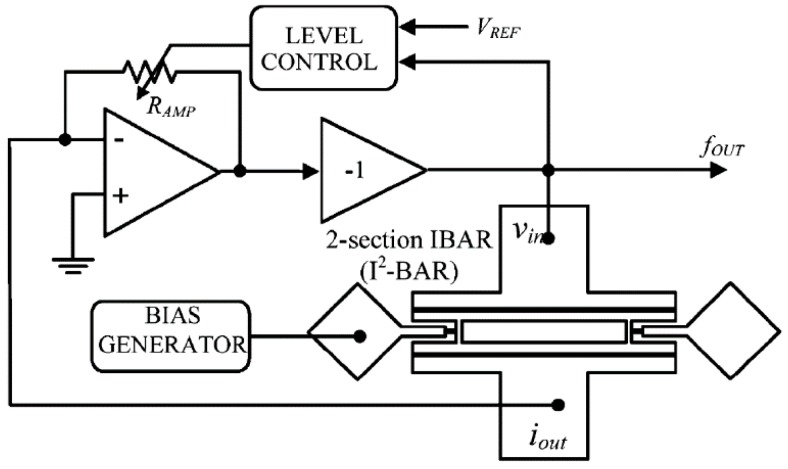
The typical trans-impedance amplifier configuration providing 0° phase shift and sufficient gain around an electrostatic resonator [241]. ^©^ 2007 IEEE. Reprinted with permission from Electronically Temperature Compensated Silicon Bulk Acoustic Resonator Reference Oscillators by Sundaresan in *J. Solid-State Circuits*, 2007.

All these specifications are challenging to fulfill simultaneously, and require carefully designed circuitry. The typical interconnection for a trans-impedance amplifier with automatic gain control used to bring a clamped-clamped beam resonator is shown in Figure 26 [241].

In this configuration, an operational amplifier is put in shunt-shunt resistive feedback to provide a trans-impedance gain of *−R*_AMP_ and a second stage provides an additional gain of −1 to provide a total positive gain with 0° phase shift and sufficient gain to offset the motional resistance of the resonator. Note that the resonator in this work has two transducer gaps, which reduces the feedthrough capacitance and thus mitigates the parallel resonance of the resonator. A level control circuit can modulate the gain by changing the feedback resistance, which is implemented with a triode transistor. Note that the use of a triode transistor can cause non-linear behavior of the circuit and detract from phase noise performance [249]. In addition, the input resistance of the shunt-shunt feedback amplifier is typically on the order of *R_F_*/*A*, where *A* is the gain of the operational amplifier in the shunt-shunt feedback. This implies that if very large gain is implemented with the amplifier or if it has insufficient gain, the input impedance may increase sufficiently to significantly load the quality factor of the resonator and deteriorate phase noise performance, as later discussed. As for resonator nonlinearity mitigation, many different amplitude limiting schemes exist, ranging from hard limiting using comparators or saturating circuits to soft limiting using variable gain amplifiers [216,231,238,250,251,252].

**Figure 27:**
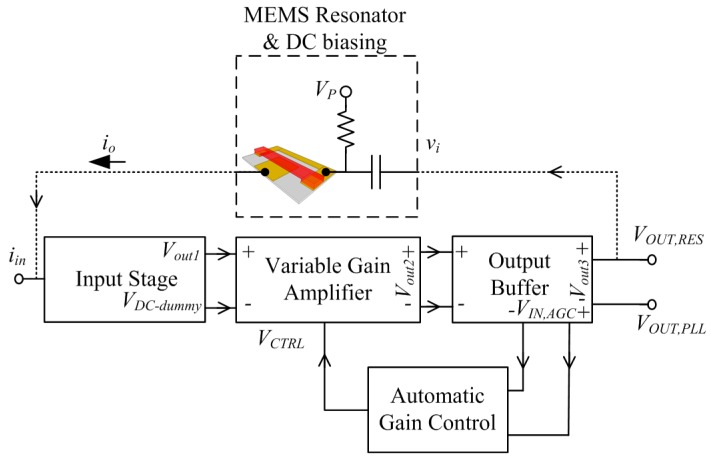
Typical trans-impedance amplifier (TIA) block diagram, showing a resonator connected in closed-loop.

In Figure 27, a topology using a trans-impedance input stage with a second voltage gain stage to provide sufficient gain is shown [215]. The advantage of splitting the gain stages into two is that more gain bandwidth can be achieved per stage to allow for series-resonant oscillation at up to 15 MHz in [208]. The shunt-shunt feedback is implemented in the variable gain amplifier. Again, an automatic gain control can regulate the gain to prevent resonator nonlinear limiting and enhance phase noise. This is implemented by varying the voltage stage gain in response to the amplitude detected at the output of the oscillator through the automatic gain control loop.

At the transistor level, the trans-impedance structure has been used both for electrostatic resonators (e.g., [201,217,231,242,245,253]) or piezoelectric resonators (e.g., [239]). Many designs utilize a regulated cascode input stage to boost the current gain and reduce the input resistance of the trans-impedance at the input, in order to reduce the Q-loading on the resonator. This is important as Q-loading in series-resonant oscillators is given by:

(17) $$Q_{L}=\frac{Q_{\text{UL}}}{1+\left(R_{i}+R_{o}\right)/R_{m}}$$

where *Q*_UL_ is the unloaded Q-factor of the resonator, *R*_m_ its motional resistance, *R_i_* the input resistance of the sustaining amplifier and *R*_o_ its output resistance. Moreover, in order to reduce the Q-loading of the resonator, all series-resonant circuits include an output stage which ensures a low output resistance, as shown in Figure 27. This can be a common-source type buffer, or a series-shunt feedback buffer circuit.

**Figure 28:**
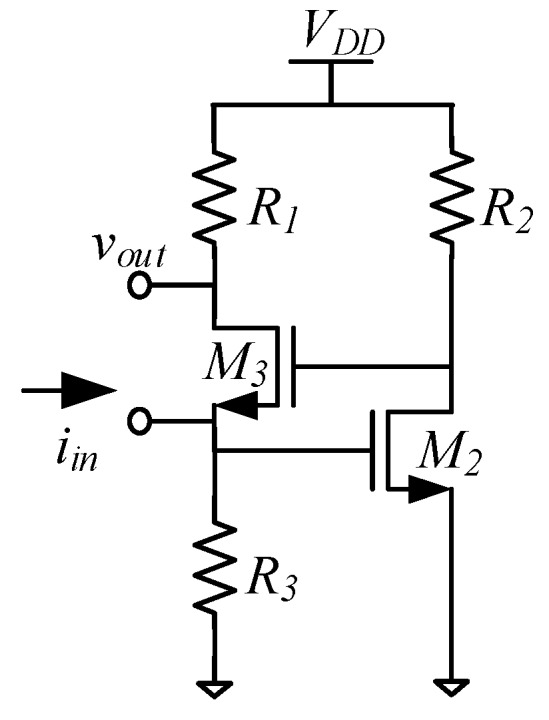
Regulated cascode trans-impedance amplifier stage.

The regulated cascode circuit is shown in Figure 28 [215] and variants of it are used in works such as [138,216,245,254]. Assuming *R*_3_ is large enough so that the signal current through it can be neglected, the low-frequency input impedance of this stage can be shown to be

(18) $$R_{i}=\frac{1}{g_{m1}\left(1+g_{m2}\left(R_{2}||r_{o2}\right)\right)}$$

It can be seen that the gain of the feedback loop increases the trans-conductance of transistor *M*_1_, and therefore lowers the input impedance of the amplifier beyond that of a traditional common-gate configuration biased at the same current. The trans-impedance gain of the stage is approximately *R*_1_, which can be selected independently to *g*_m1_ and *g*_m2_ with appropriate transistor sizing. This is in contrast to the shunt-shunt amplifier which imposes relationship between the achievable gain and the input resistance, as was previously discussed. To achieve a large gain, the size of *R*_1_ must be maximized, but the voltage drop across it must be small enough to keep *M*_1_ in saturation mode. This reveals the main advantage of the *g*_m_-boosted topology, compared to a simple common-gate amplifier. A small bias current can flow through *M*_1_ and *R*_1_, allowing for a large trans-impedance gain, while the feedback provided by *M*_2_ and *R*_2_ boosts the trans-conductance of *M*_1_ such that the input impedance remains small. Note that the resistors can be implemented as triode transistors as well, but care should be given to the 1/*f* noise performance in that case.

**Figure 29:**
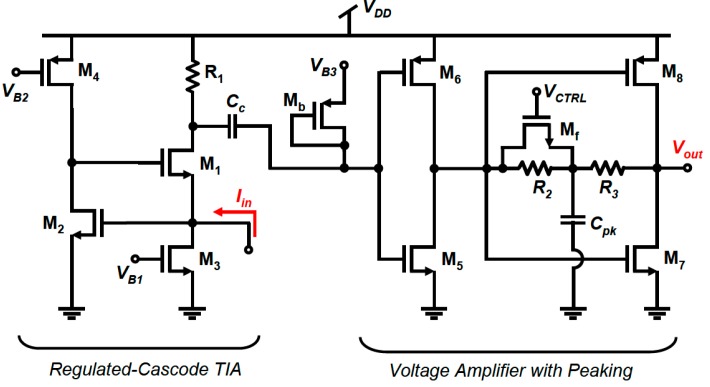
Voltage gain stage used to increase the gain of the regulated cascade amplifier [253]. ^©^ 2012 IEEE. Reprinted with permission from A 1.57 mW 99 dBΩ CMOS transimpedance amplifier for VHF micromechanical reference oscillator by Li in Proceedings of the IEEE International Symposium on Circuits and Systems, 2012.

Typically following the regulated cascode is a variable voltage gain amplifier in shunt-shunt feedback using a triode transistor to vary the gain such as in [253], and shown in Figure 29 [253] following a regulated cascode input stage using two triode transistors *M*_3_ and *M*_4_. The voltage gain amplifier this case is composed of two inverters (*M*_5_, *M*_6_ and *M*_7_, *M*_8_), with the second inverter in shunt-shunt feedback allowing for controllable gain through the biasing of transistor *M*_f_. Capacitor *C_pk_* is included in this case in order to provide a peaking zero in the frequency response which extends the bandwidth of the amplifier and ensures a sufficient bandwidth to meet the 0° phase condition for series-resonant oscillation. Note that the first inverter in the voltage amplifier can be difficult to bias properly because of the sensitive nature of the inverter input node to DC bias. As such in [138], a less sensitive common source voltage gain stage is used before the shunt-shunt feedback tunable voltage stage.

**Figure 30:**
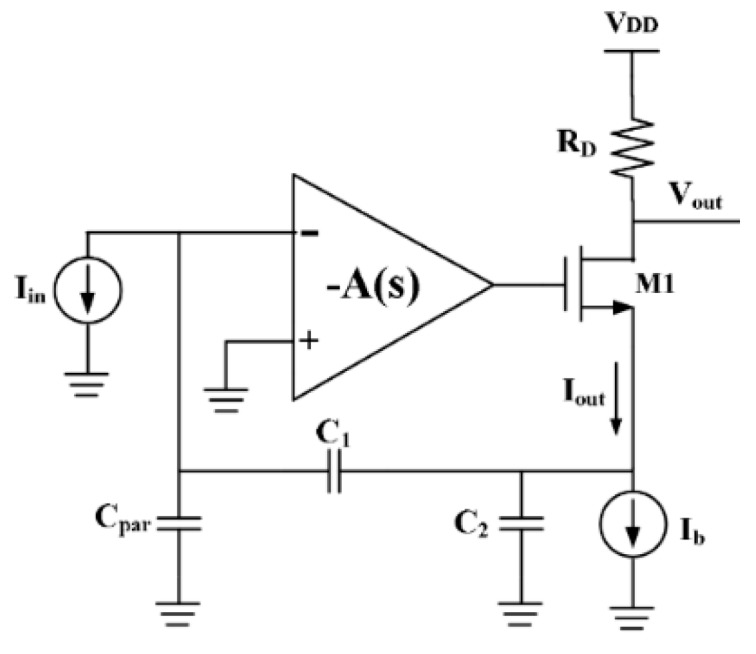
Capacitive sustaining amplifier circuit [254]. ^©^ 2014 IEEE. Reprinted with permission from CMOS 0.18 μm standard process capacitive MEMS high-Q oscillator with ultra low-power TIA readout system by Kuo in Proceedings of the IEEE Sensors, 2014.

Another amplifier structure which has been used is the capacitive feedback trans-impedance amplifier structure [218,255,256]. This structure is shown in Figure 30 [254] and includes an operational amplifier which is configured with capacitive feedback, allowing for a very large gain and very low noise since the capacitor element does not contribute any noise to the circuit, unlike the feedback resistor used in shunt-shunt configurations. This topology requires careful design of the frequency response of the circuit to ensure proper phase shift for oscillation.

**Figure 31:**
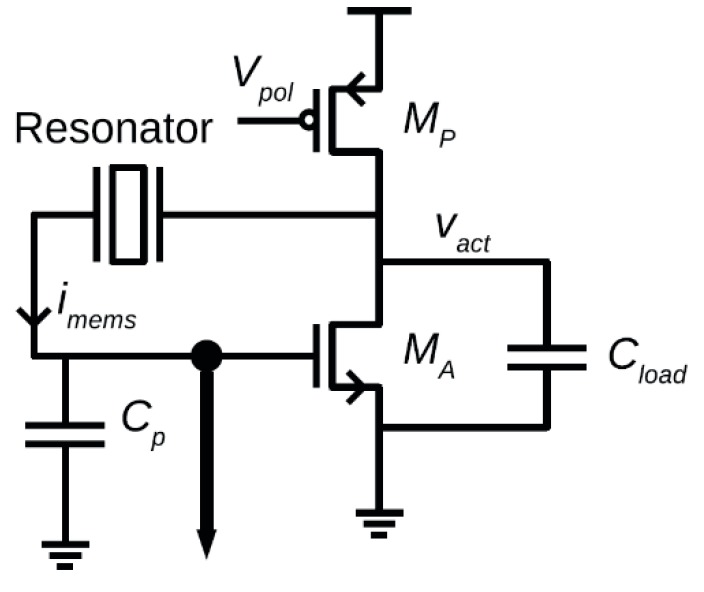
Typical Pierce oscillator configuration [214]. Reproduced with permission from Arndt et al., *Sens. Actuators Phys.*; published by Elsevier, 2011.

In addition, the Pierce oscillator structure shown in Figure 31 [214], often used in quartz oscillators, has also been used in MEMS resonator-based oscillators. In this architecture, one transistor is used to provide negative gain, and load capacitance ensures minimal Q-loading of the resonator and optimal phase shift. This configuration utilizes parallel resonance of the resonator to attain the additional phase shift required to attain oscillation. The Pierce structure, due to its reduced achievable gain is mostly used in piezoelectric MEMS resonator-based oscillators (e.g., [163,215,257,258]). However, this topology is less used in electrostatic resonators, as they generally have too large of a motional resistance to be overcome by the one transistor stage Pierce structure. Notably, some works involving electrostatic resonators have been able to achieve Pierce-type oscillators by using relatively small electrostatic gap resonators in order to reduce the motional resistance sufficiently (i.e., 50 nm in [249]). In that regard, works have shown methods of reducing the electrostatic gap-size after fabrication using static electrostatic actuation of the resonant structure towards the drive and sense electrodes, potentially allowing for more widespread use of the Pierce structure [259,260]. MEMS oscillators can also operate using parallel resonance though a higher gain inverting amplifier (e.g., current starved inverter in [201]) and capacitive elements to provide additional phase shift.

It is important to note that while some oscillators feature single-ended sustaining amplifier structures, many also are implemented using differential structures, in order to reduce the impact of common-mode noise, and take advantage of resonators having differential resonant modes which can result in better phase noise performance (e.g., [256,260]).

#### 8.2.5. Nonlinear Oscillators

Some efforts have been carried out to operate MEMS resonators in their non-linear Duffing regime in order to achieve more than 1/*f*^2^ phase noise filtering, and significantly improve the close-in phase noise performance of MEMS oscillators [220]. Work in [220] and all of the previously discussed series and parallel resonant oscillators are harmonic in nature, such that a resonator, acting as a bandpass filter is put within a positive feedback loop to generate an oscillation at a frequency within the resonator’s series or parallel resonance frequencies.

Recently, nonlinear oscillators, such as the parametric oscillator, originally introduced in RF and microwave applications (see [259]), have been applied to MEMS resonators [224,260,261,262,263]. The simplest parametric oscillators operate on the modulation of a characteristic in a pumping loop at twice the required resonator resonant frequency [260]. When doing so, the resonator will generate a signal through its non-linearity that will provide the required output frequency. The fact that no electronics operating at the resonant frequency are required to close a harmonic loop around the resonator allows for these oscillators to feature low close-in phase noise performance (i.e., within the resonator bandwidth) since the electronic noise is not dominating at the resonant frequency [224,263,264].

**Figure 32:**
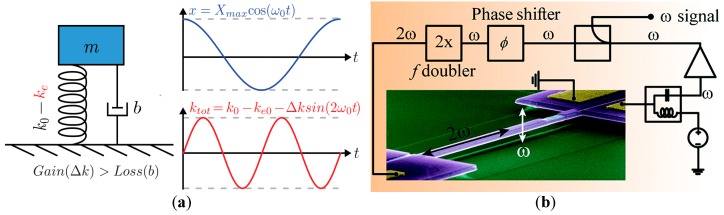
(**a**) Parametric pumping of a MEMS electrostatic resonator [223]; ^©^ 2014 IEEE. Reprinted with permission from A micromechanical parametric oscillator for frequency division and phase noise reduction by Rocheleau in the Proceedings of International Conference on Micro Electro Mechanical Syst., 2014; and (**b**) a typical parametric oscillator loop used with a nanoscale resonant beam [261]. Reproduced with permission from Villanueva et al., *Nano Lett.*; published by American Chemical Society, 2011.

Electrostatic resonators are particularly well-suited for harmonic oscillators, as their spring constants can be modulated using a variation of their bias voltages, such as shown in Figure 32a [223] and Figure 32b [261]. This can be done by a specially designed oscillator operating at double the resonator resonator’s frequency [223], or by putting the resonator within a loop that includes a frequency-doubler and start-up source [261]. Parametric oscillation can also be carried-out with piezoelectric resonators, but the higher linearity of the piezoelectric transducer has required the use of a voltage varying capacitor (i.e., a varactor), in the parametric amplifier to generate the harmonic behavior [262].

This approach notably has for advantage of allowing lower-Q-factor resonators to be used and deliver performance akin to higher-Q devices, allowing for the design using moderate Q-factor resonator which may have power handling advantages that can reduce far offset phase noise performance further, while providing adequate close-in phase noise performance through a parametric oscillator configuration.

### 8.3. Sensing

Operation of resonant sensors is based on transforming energy in from one domain to a change in the resonant frequency of a device. The change in resonant frequency can be detected by sweeping the frequency of an excitation signal around the resonant frequency of the device. A more practical method is to place a two-port resonant device within the feedback loop of a properly designed electronic circuit to form an oscillator. In this case, measurements of the quantity of interest will be carried through monitoring of the output frequency of the oscillator circuit. Resonant sensors are among the most sensitive and precise devices for many applications. This in great part is due to the variety of existing techniques for fast, precise, and accurate measurement of the frequency or period of a signal. It is relatively simple to measure the frequency of a signal with sub-ppm levels of precision while achieving a similar level of precision on a voltage or current measurement is typically a challenge. Accurate frequency references, from crystal oscillators to atomic clocks, offer better stability than typical voltage or current references. On the other hand, the signal from a resonant sensor is quasi-digital since an analog-to-digital converter is not needed to measure a frequency. As the information is embedded in frequency rather than amplitude, the sensor signal is immune to noise and interference.

Recalling that $\omega_{0}=\sqrt{K_{eff}/M_{eff}}$, it can be seen that a shift in the resonant frequency of a structure is a consequence of a change in the effective mass or the effective spring constant of the structure:

(19) $$\Delta f_{0}=\frac{f_{0}}{2}\left(\frac{\Delta K_{eff}}{K_{eff}}-\frac{\Delta M_{eff}}{M_{eff}}\right)$$

where $\Delta K_{eff}$ and $\Delta M_{eff}$ are disturbances in the effective stiffness and mass of the structure, respectively. The challenge, therefore, is the proper design of mechanical coupling structures to transform the information from the desired physical domain to a change in dynamic properties of a micromechanical device. Consequently, the design of resonant sensors involves the design of a (high-Q) resonator as well as coupling mechanisms that convert the quantity of interest into a mass or stiffness disturbance. The changes in stiffness are usually a consequence of developing internal stresses within the structure of the device. Obvious applications of resonant sensors are thus mass or strain sensing.

Changes in resonant frequency can be measured in different ways. The most straightforward method, especially in a laboratory setting, is to sweep the frequency of the excitation signal around resonant frequency and monitor the changes in resonant frequency in response to disturbances. Another technique is to excite the resonator with a fixed stable signal at a frequency that is slightly different (typically higher) than the resonant frequency of the device. In this case, changes in the resonant frequency will be converted to changes in the amplitude of the output signal and can be related back to the measurand. A technique more suitable for general applications is to place the resonator in the feedback loop of an oscillator circuit and monitor its output frequency. In all these cases, higher quality factors result in better resolutions for the sensor. Therefore, maximizing the resonant sensor quality factor is another design and operation requirement. It should be noted that too high a Q can lead to large resonance amplitudes, and hence, make the resonator prone to nonlinearities.

It is noteworthy that, as can be seen from Equation (5), changes in quality factor can also affect the resonant frequency of a device. However, quality factor of microdevices is often quite large and varies significantly from device to device. As such, both precision and accuracy advantages may be sacrificed to a great extent. If the parameter of interest affects the quality factor, changes in signal amplitude are typically easier to measure than changes in the resonant frequency. Additionally, a high quality factor often reduces the sensitivity of the resonator response to changes in quality factor.

#### 8.3.1. Resonant Sensors Based Changes in Effective Mass

Resonant mass sensing, also known as gravimetric sensing, has been a well-known application of resonators. The change in the effective mass of the structure can be due to settlement of particles and objects on the resonators surface, deposition of thin films, or absorption of material into films on the surface of the device. Resonant mass sensors have therefore been used to detect particle concentration, deposition rate, chemical sensing, and bio-sensing. As can be inferred from Equation (9), to obtain a high mass sensitivity, one would like to use a device with a high resonant frequency $f_{0}$, with a small effective mass, $M_{eff}$. Reducing device dimensions usually achieves both of these goals at the expense of complexity of input/output coupling to the device.

**Figure 33:**
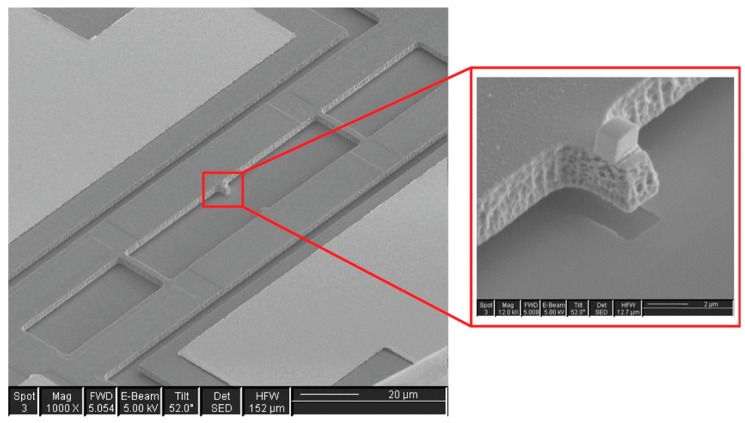
A resonant mass sensor based on coupled resonators. Note the addition of platinum sheets used for characterization of the sensor [269]. ^©^ 2016 IEEE. Reprinted with permission from Improving sensitivity of resonant sensor systems through strong mechanical coupling by M.S. Hajhashemi in *J. Microelectromech. Syst.*, 2016.

Beam based structures, such as cantilevers and bridges, offer a simple structure, straightforward excitation, and various detection possibilities [48,265,266,267,268,269,270,271]. Figure 33 is an SEM image of a bridge-based mass sensor where sheets of platinum were deposited for controlled characterization of the device. Even single carbon nanotubes can be used as super-sensitive cantilever-based mass sensors [272,273]. To use these devices, the beam resonator is excited in its flexural, typically out-of-plane, mode of vibration using piezoelectric or electrostatic actuation. The resonant frequency of the beam is then monitored using optical or electrical techniques as to quantify the mass added to the structure. For flexural vibrations of a beam, the contributions of different segments of the structure to its effective mass vary depending on the beam geometry and the relative amplitude of vibration (see Equation (19)). The location of added mass onto the beam is thus another factor that affects the amount of shift in the resonant frequency and must be taken into account in such studies [274,275].

To go beyond the capabilities of a simple cantilever structure without dealing with complexities of driving and sensing signals at the nano-scales, one can take advantage of bulk resonant modes of structures. Bulk mode resonators, in general, offer higher resonant frequencies for similar dimensions as flexural devices. Furthermore, they often have higher quality factors, which improves the resolution of the sensors [274]. The structure of a bulk mode resonators is typically simple to reduce the effects of spurious modes and is usually based on a beam, square or circular plate, or a ring. Any of surface acoustic wave [276,277,278], thickness shear [279,280], bulk acoustic wave [281,282], or extensional modes [283,284] can be selected for mass sensing among several other modes of vibration [283]. The selection of the mode shape is usually influenced by fabrication capabilities and the desired transduction mechanisms.

One of the main applications of gravimetric sensors is chemical sensing, particularly in gaseous phase [284]. In such applications, a thin film that selectively binds to the target chemical is applied to the surface of the resonator. During the device operation, the analyte is adsorbed onto or absorbed into this sensitive layer, causing an increase in the effective mass of the structure, among other potential effects. For instance, molecules absorbed into the film often produce a stress at the surface of the structure, which besides affecting the resonant frequency of the resonator, modifies the surface losses in the structure and alter the quality factor of the resonator. The resonator, on the other hand, is usually placed in an oscillator loop and the changes in its resonant frequency are monitored. For a perfectly selective film, the changes in the effective mass can be directly related to the concentration of the particular chemical in the environment. In practice, however, various other phenomena may affect the resonant frequency of the device including variations in temperature, humidity, and other molecules that may also bind to the film. Cantilevers and bridges are among the more common types of resonators that are used for gravimetric gas sensing in gases [285,286,287,288,289]. The simple structure of these resonators makes it possible to fabricate high performance devices even when the manufacturing process is not optimal [290,291,292]. The large surface area of quartz microbalances or surface acoustic wave resonators makes them popular choices for gas sensing applications as the focus is placed on the thin film [293,294,295,296]. Gravimetric sensors based on nano-resonators are capable of detecting a single molecules of target analyte. Arrays of gravimetric gas sensors have been used in multi-gas sensors systems, also known as electronic-noses [297,298,299].

**Figure 34:**
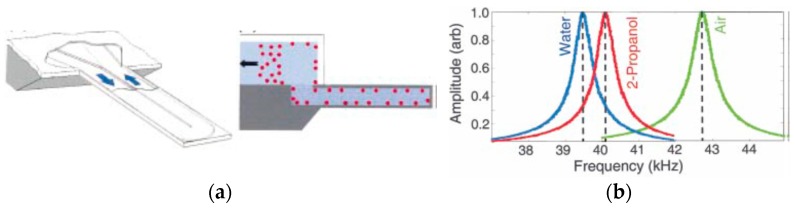
(**a**) Damping in liquid phase resonant mass sensors are reduced by using suspended microfluidic channels as resonators; (**b**) Analytes are detected based on their mass density difference relative to the surrounding solution. [43]. Reproduced with permission from T.P. Burg, *Appl. Phys. Lett.*; published by AIP, 2003.

Resonant mass sensors are also used for the detection of biomolecules. In this case, the active surface of the resonator is coated with a protein that selectively binds to the target biomolecule. However, as most biological sensing needs to be carried within biofluids, most resonant modes are damped so heavily that they could no longer be used as gravimetric sensors. The resonant modes that can still result in high quality factors often rely on in-plane movements of the resonator relative to the liquid surface [148,274,279,300,301,302]. It is also possible to build micro- or nano-channels within the structure of the resonator (see Figure 34) [45]. Once the inside walls of these channels are activated, the biosample is flown through the channels. Target biomolecules in the sample bind to the channel walls, increasing the effective mass of the resonator. This technique allows for the use of various resonators at the expense of increased fabrication complexity.

#### 8.3.2. Resonant Sensors Based Changes in Effective Stiffness

The stiffness of most mechanical structures is a function of the stress applied to them. A familiar example is a guitar string whose natural frequency increases with tensile stress. Most resonant sensors employ the flexural resonant modes of a structure. This in part is due to the fact that it is fairly simple to couple an input stress onto a flexural spring without affecting other resonator parameters especially its quality factor. For a clamped-guided beam, a common element in MEMS structures. The effective flexural spring constant of such a beam under axial force T (positive for tensile forces) is given by [302]:

(20) $$k_{b}=\frac{\gamma T}{\gamma L-2\text{tanh}\left(\frac{\gamma L}{2}\right)}\approx \frac{12EI}{L^{3}}+\frac{6T}{5L}$$

where E is the Young’s modulus of the material, L and I are the length and the second moment of inertia of the beam spring, respectively, and γ=TEI. As can be seen, the flexural spring constant changes linearly for small axial forces (stiffens for tensile stress and softens for compressive). The extreme sensitivity of resonant sensors allows for the detection of strains on the order of pico- to nano-strains, and hence, a viable mechanism for detection of various phenomena. To maintain high quality factor, most resonant sensors are operated under vacuum. This encapsulation creates challenges for the coupling of the measurand to the resonator structure.

Much like mass sensing, strain sensing is an obvious application of resonant sensors. A strain sensor, in its simplest form, is anchored at two locations which undergo some relative displacement during the device operation. The anchors transfer this strain to the resonator structure, and consequently, affect its resonant frequency [303,304,305,306].

**Figure 35:**
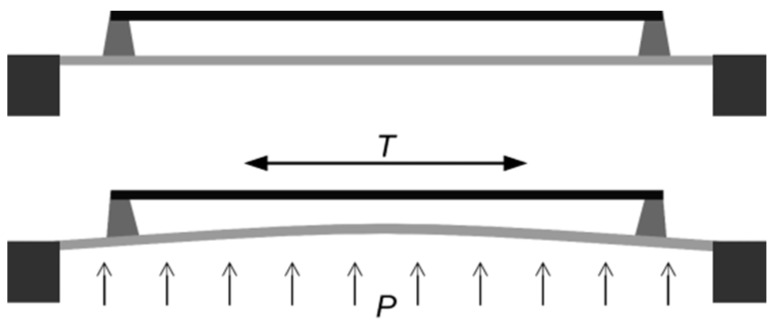
Generation of axial strains in bridges anchored to a flexible membrane due to pressure.

**Figure 36:**
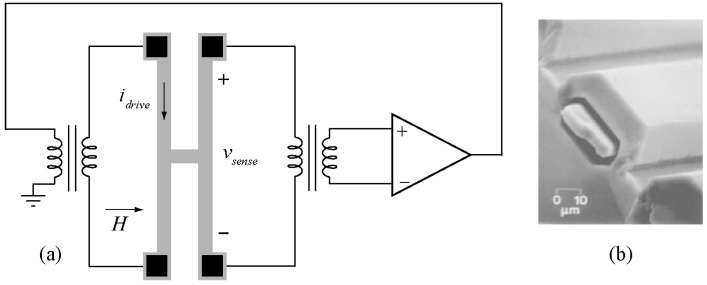
The resonant pressure sensor by Ikeda et al. [311]: (**a**) the schematic of the resonator and circuitry around it and (**b**) cross-sectional view of one of the beams inside the cavity and the encapsulation film. ^©^ 1998 IEEE. Reprinted with permission from Silicon Micromachined Vacuum Encapsulated Resonant Pressure Sensors by K. Ikeda in Proceedings of International Microprocesses and Nanotechnology Conference, 1998.

Pressure sensors were among the early examples of resonant sensors. The design of these devices is based on anchoring a resonator to one side of a membrane that would deflect under pressure. The membrane deflections would then cause an axial strain onto the resonator, ultimately causing a change in its resonant frequency (see Figure 35). The required high quality factor was achieved through providing a stable, low-pressure ambient on the resonator side of the membrane [307,308,309,310,311]. Figure 36 shows such a device where coupled bridge resonators were enclosed in a micro-cavity and anchored to the top of a flexible membrane [312,313]. The device was placed within a fixed magnetic field produced by a permanent magnet. A current is used to produce a Lorentz force on one arm of the device whose dynamic deflections are picked up using the electromotive voltage produced across a coupled beam. The device was then placed within the feedback loop of an oscillator circuit.

**Figure 37:**
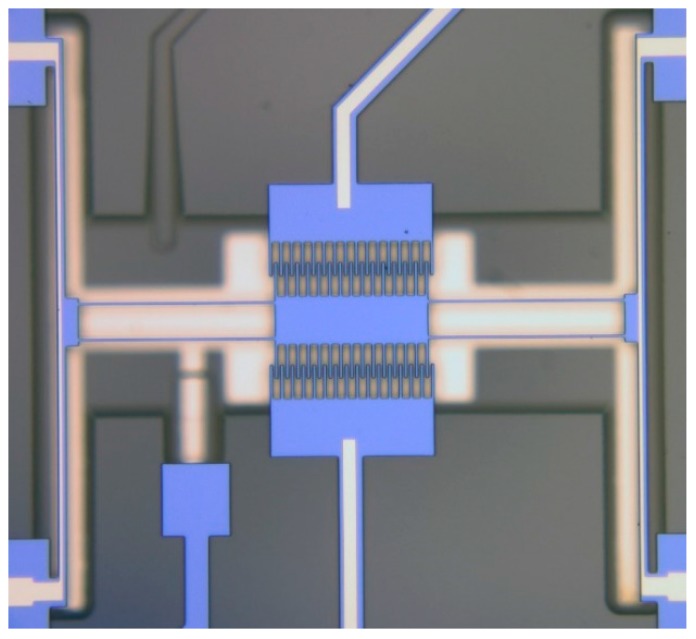
A resonant micromachined magnetic field sensor [315]. A DC current is passed through the two metal coated cross-bars which generates a Lorentz force that results in an axial force on the beam springs of the electrostatic resonator.

Several resonant magnetic field sensor designs have also been proposed [304,314,315,316]. In a typical design, the Lorentz force on a current-carrying beam is used to generate a stress on a resonator, whose resonance frequency then changes accordingly. Figure 37 illustrates a sample design where the Lorentz force on two cross-bars was transferred axially to an electrostatic resonator [315]. Thanks to the inherent advantages of resonant sensing, resonant magnetic sensors typically offer a high resolution and dynamic range without the need for any special fabrication steps or materials. Some magnetic sensors designs employ resonance to enhance the sensor response, but their output is based on a change in signal amplitude rather than frequency [317,318,319].

**Figure 38:**
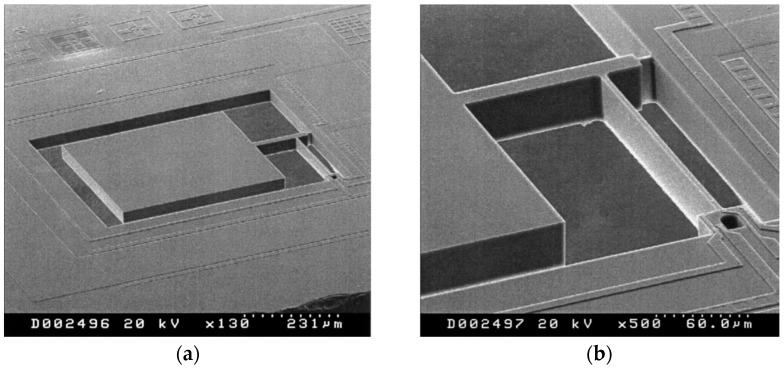
A resonant accelerometer: (**a**) The structure was brought under resonance using a thermal actuator embedded at the base of connector beam (**b**). Strains were measured using doped piezoresistors [321]. Reproduced with permission from Aikele, M. *Sens. Actuators Phys.*; published by Elsevier, 2001.

Linear inertial forces on structures can also be coupled to a resonator structure to realize resonant accelerometers [320,321,322]. Figure 38 is an SEM image of a resonant accelerometer where displacements of proof mass were measured through changes in the resonant frequency of the connector beam [321]. It is also possible to measure the proof mass displacements through monitoring the change in the electrostatic spring constant in electrostatic resonators [322]. Even though resonant accelerometers are capable of achieving very high resolutions, their relatively slow response times makes their applications limited to special cases. Vibratory gyroscope designs are also based on microresonator structures. However, in typical designs, two resonators or two vibration modes of a single resonator are employed and the measurement of rate is based on change in signal amplitudes [323,324,325,326,327].

Structures can be designed to convert a Columbic force to an axial stress. The Columbic force can be generated from a field produced by an electric charge. This concept has been employed design and fabricate micromachined resonant charge sensors [328,329].

### 8.4. Radio-Frequency Systems

MEMS resonators have the potential to be used in RF systems as filters in various locations of the transceiver chain or as oscillator core elements to directly generate RF carriers or generate reference frequencies for LC RF oscillators in RF frequency synthesizers [7,86,330,331].

**Figure 39:**
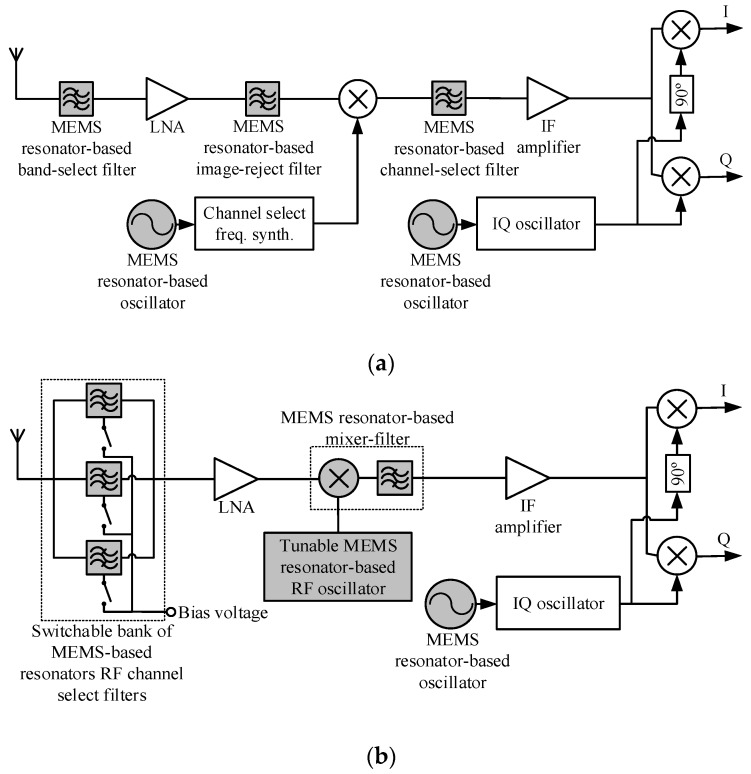
(**a**) Super-heterodyne architecture with off-chip components replaced by integrated MEMS resonators; and (**b**) a resonator-based receiver architecture.

This is illustrated in Figure 39a, where MEMS resonators could replace the band-select, image-reject, or channel-select filters. They could also be used in the channel-select phase-locked loop as frequency references. The very high quality factor and GHz-range resonant frequencies attainable by MEMS resonators can also allow for receiver architectures that are not possible with other filter technologies. This is illustrated in Figure 39b, where electrostatic MEMS resonators resonating at RF frequencies can be used to filter the channel directly before the low-noise amplifier (LNA) [7,11,332,333,334]. Moreover, the MEMS resonators can be used as mixer-filters by leveraging their non-linearity to provide tuned carrier down-conversion [333], and the RF local oscillator can be implemented without a phase-locked loop [210]. Besides the inherent area and cost savings, the architecture in Figure 39b can be used to trade off high-Q for power consumption [330] as channel selection directly carried-out at RF yields a substantial advantage: the dynamic range and linearity requirements of the LNA and mixer in the receive path can be reduced. This is because high power interferers can be rejected not only at the band-level but also at the channel-level, only leaving the channel of interest to be amplified. Moreover, an RF-level channel-filter can relax the phase noise requirements of the RF oscillator relative to adjacent channel interferers, as these channels are already attenuated before the down-conversion.

With regards to filtering, MEMS resonators can be electrically viewed as bandpass filters with very small bandwidths, having center frequencies determined by their intended resonant mode. MEMS resonators can also be combined in order to create higher order bandpass filters which can potentially be integrated into transceiver front-ends by replacing costly, off-chip, narrow-bandwidth filters. In order to achieve a bandpass filtering function with sufficient bandwidth, MEMS resonators need to be coupled together as their quality factors are generally too high to provide a wide enough bandwidth standalone. This coupling between resonators to achieve the required flat enough passband can be done mechanically (e.g., [86,210,335,336,337,338]) using springs, typically implemented through torsional, flexural or extensional beams, or electrically using intrinsic capacitances (e.g., [64,88,141,339]). Band stop filters have also been proposed [338] and wideband filters have also been demonstrated [175].

An obstacle to the deployment of MEMS resonators as filters in RF systems is their insertion losses that can be on the order of several dBs [11], causing too much noise figure degradation if used before the low-noise amplifier. Moreover, the motional resistance of electrostatic MEMS resonators is usually on the order of several kilo ohms, which requires matching networks to be implemented in order to ensure correct filter terminations in 50 Ω RF systems [11]. This can be circumvented by using piezoelectric MEMS resonators (e.g., [64,141,142,340,341]) that can have lower motional resistances on the order of 50 Ω. Moreover, resonator arraying can mitigate the large motional resistance in the case of electrostatic resonators [79]. Other works include a post filter trans-impedance amplifier to increase the signal strength after the filter, at the cost of increased power consumption and no noise figure benefit [337,338].

Regardless of these challenges, piezoelectric bulk acoustic wave (BAW) MEMS resonators have successfully been demonstrated in receivers [15,211] to perform PLL-free frequency synthesis and channel selection at RF.

## 9. Conclusions

In this review paper, we have endeavored to illustrate the diversity of progress made in the field of micromachined resonators and the divergence in approaches and applications that have been pursued over the last three decades. We began with a description of the basic model and properties of a generic micromachined resonator as the unifying and starting spring board from which the various key aspects of interest pertaining to resonator design and implementation were subsequently described. We have seen that there exists not only a great diversity in the vibration modes reported in the literature but also a plethora of approaches in the fabrication technology as well as materials. While the field of started off with single-crystal silicon and polysilicon processing dominating the literature, this review has shown the great diversity of process technologies that illustrate the divergence of the field today represented by piezoelectric thin films (e.g., AlN, PZT, ZnO) and bulk materials (e.g., LiNbO_3_) as well as micromachining in standard CMOS fabrication technology. We have also presented the variety of transduction mechanisms that have been actively employed during the progress of this field in addition to capacitive transduction that was the most common method in the early days of development. This variety of transduction mechanisms includes recent findings suggesting that thermal actuation has a place for actuation in high frequency resonators. While analytical modeling of damping mechanisms remains highly complex and out of reach, numerical methods for capturing specific energy loss mechanisms such as anchor loss have become highly popular among designers. Advancement in the field of microresonators has gone beyond the resonator itself to include interface electronics such as in the case of implementing oscillators. We have described key circuit topologies that have been employed to realize MEMS oscillators, as well as developments in addressing the issue of temperature compensation. With respect to applications, we have shown how resonators constitute key elements in radiofrequency frequency control, sensing (mass and force) and timing references. We envisage that the range of applications for resonators will expand further with increasing advancement in their fabrication, design and analysis.

## Figures and Tables

**Table 1:** Correspondence between electrical and mechanical domains.

Mechanical Domain	Electrical Domain
Force, F	Voltage, V
Velocity, $\dot{x}$	Current, I
Displacement, x	Charge, q
Compliance, 1K	Capacitance, C
Mass, M	Inductance, L
Damping, ζ	Resistance, R
